# Deep learning for predicting invasive recurrence of ductal carcinoma *in situ*: leveraging histopathology images and clinical features

**DOI:** 10.1016/j.ebiom.2025.105750

**Published:** 2025-05-28

**Authors:** Shannon Doyle, Esther H. Lips, Eric Marcus, Lennart Mulder, Yat-Hee Liu, Francesco Dal Canton, Timo Kootstra, Maartje M. van Seijen, Ihssane Bouybayoune, Elinor J. Sawyer, Alastair M. Thompson, Sarah E. Pinder, Clara I. Sánchez, Jonas Teuwen, Jelle Wesseling, Grand Challenge PRECISION Consortium, Jelle Wesseling, Jos Jonkers, Jacco van Rheenen, Esther H. Lips, Marjanka Schmidt, Lodewyk F.A. Wessels, Proteeti Bhattacharjee, Alastair Thompson, Serena Nik-Zainal, Helen Davies, Elinor J. Sawyer, Andrew Futreal, Nicholas Navin, E. Shelley Hwang, Fariba Behbod, Daniel Rea, Hilary Stobart, Deborah Collyar, Donna Pinto, Ellen Verschuur, Marja van Oirsouw

**Affiliations:** a Division of Radiation Oncology, Netherlands Cancer Institute - Antoni van Leeuwenhoek, Amsterdam, the Netherlands; b Informatics Institute, University of Amsterdam, Amsterdam, the Netherlands; c Division of Molecular Pathology, Netherlands Cancer Institute - Antoni van Leeuwenhoek, Amsterdam, the Netherlands; d Department of Medical Imaging, Radboud University Nijmegen, Nijmegen, the Netherlands; e Department of Pathology, Netherlands Cancer Institute – Antoni van Leeuwenhoek, Amsterdam, the Netherlands; f Department of Pathology, Leiden University Medical Center, Leiden, the Netherlands; g School of Cancer & Pharmaceutical Sciences, King's College London, UK; h Department of Surgery, Baylor College of Medicine, Houston, TX, USA

**Keywords:** Deep learning, Ductal carcinoma in situ, Multiomic integration, Risk prediction

## Abstract

**Background:**

Ductal Carcinoma *In Situ* (DCIS) can progress to ipsilateral invasive breast cancer (IBC) but over 75% of DCIS lesions do not progress if untreated. Currently, DCIS that might progress to IBC cannot reliably be identified. Therefore, most patients with DCIS undergo treatment resembling IBC. To facilitate identification of low-risk DCIS, we developed deep learning models using histology whole-slide images (WSIs) and clinico-pathological data.

**Methods:**

We predicted invasive recurrence in patients with primary, pure DCIS treated with breast-conserving surgery using clinical Cox proportional hazards models and deep learning. Deep learning models were trained end-to-end with only WSIs or in combination with clinical data (integrative). We employed nested k-fold cross-validation (k = 5) on a Dutch multicentre dataset (n = 558). Models were also tested on the UK-based Sloane dataset (n = 94).

**Findings:**

Evaluated over 20 years on the Dutch dataset, deep learning models using only WSIs effectively stratified patients into low-risk (no recurrence) and high-risk (invasive recurrence) groups (negative predictive value (NPV) = 0.79 (95% CI: 0.74–0.83); hazard ratio (HR) = 4.48 (95% CI: 3.41–5.88, p < 0.0001); area under the receiver operating characteristic curve (AUC) = 0.75 (95% CI: 0.70–0.79)). Integrative models achieved similar results with slightly enhanced hazard ratios compared to the image-only models (NPV = 0.77 (95% CI 0.73–0.82); HR = 4.85 (95% CI 3.65–6.45, p < 0.0001); AUC = 0.75 (95% CI 0.7–0.79)). In contrast, clinical models were borderline significant (NPV = 0.64 (95% CI 0.59–0.69); HR = 1.37 (95% CI 1.03–1.81, p = 0.041); AUC = 0.57 (95% CI 0.52–0.62)). Furthermore, external validation of the models was unsuccessful, limited by the small size and low number of cases (22/94) in our external dataset, WSI quality, as well as the lack of well-annotated datasets that allow robust validation.

**Interpretation:**

Deep learning models using routinely processed WSIs hold promise for DCIS risk stratification, while the benefits of integrating clinical data merit further investigation. Obtaining a larger, high-quality external multicentre dataset would be highly valuable, as successful generalisation of these models could demonstrate their potential to reduce overtreatment in DCIS by enabling active surveillance for women at low risk.

**Funding:**

10.13039/501100000289Cancer Research UK, the Dutch Cancer Society (KWF), and the 10.13039/501100002999Dutch Ministry of Health, Welfare and Sport.

**Research in context**

**Evidence before this study**

Despite numerous clinical, pathological and radiological factors showing association with the risk of invasive breast cancer recurrence after treatment of DCIS, none of these biomarkers have resulted in clear clinical utility. In order to assess the state of research using AI-based prognostication in DCIS, a comprehensive PubMed literature survey was conducted on April 22, 2024. This search, conducted without language or date restrictions, employed the query (“recurrence” OR “prognosis” OR “prognostication” OR “risk stratification” OR “prediction model” OR “decision support”) AND (“DCIS” or “ductal carcinoma in situ”) AND (“deep learning” OR “artificial intelligence” OR “machine learning”). We systematically assessed 23 original research studies out of the 28 search results. Few studies applied deep learning or machine learning to predict outcomes in DCIS; four utilised histopathology images, one mammography, and three immunofluorescence microscopy.

Among the histopathology studies, only one applied end-to-end deep learning, however, this was a proof-of-concept study (training dataset n = 67; validation dataset n = 66), and diverged from ours in its study population, including DCIS patients with invasion and any type of treatment, and research question, predicting invasive and in-situ recurrence in a short follow-up of three years. Two studies employed machine learning on pathomics features, with one predicting DCIS recurrence as invasive breast cancer, while the other predicted risk according to Oncotype DX category, which correlates with both invasive and in-situ recurrence. Another study used machine learning to forecast invasive recurrence for DCIS based on clinical and radiological features.

No studies developed deep learning models to stratify patients with primary, pure DCIS by the risk of invasive recurrence, nor did any analyse a population-based cohort for model development and testing, underscoring the relevance of this research.

**Added value of this study**

We developed deep learning models able to stratify patients with primary, pure DCIS based on invasive breast cancer risk using a population-based cohort of 558 patients from the Netherlands with a 20-year median follow-up. Our computational pipeline uses whole-slide images (WSIs) alone or in conjunction with clinico-pathological variables, without the need for any manual intermediary steps. Our results show that the prediction of DCIS as ipsilateral invasive breast cancer is feasible solely using whole-slide images, and the integration of clinico-patholigical factors merits further investigation.

Our study also demonstrates that deep-learning models exhibit superior performance in stratifying patients into different risk groups compared to clinical models based on Cox proportional hazard modelling. Furthermore, our study highlights the necessity of large and high-quality datasets for validation, since we encountered difficulties validating our models on a small multicenter cohort from the UK (n = 94) with compromised WSI quality. To promote the dissemination of our research, we have released the source code under open-source licence with publication.

**Implications of all the available evidence**

Our results demonstrate the feasibility of a deep learning model distinguishing patients with DCIS by their risk of developing an ipsilateral invasive breast cancer recurrence directly from WSIs. This type of classifier could be seamlessly integrated into the clinical workflow without the need for additional tests or pathology work. Additionally, our results suggest further exploring the effectiveness of multi-modal learning and enhancing generalisation capacity in these models. Upon successful external validation, the model presented herein could lead to improved clinical-decision, particularly favouring less intensive treatment for women predicted to be at low-risk, preserving the quality of life of many women, and thus optimising patient care.

## Introduction

Ductal carcinoma *in situ* (DCIS) is a breast pathology frequently detected by population-based breast cancer screening. While DCIS can progress to ipsilateral invasive breast cancer (iIBC),^1^ at least 3 out of 4 DCIS lesions will never progress or cause any symptoms.^2^, ^3^, ^4^ Because we cannot reliably distinguish non-progressive from potentially progressive DCIS, women with DCIS are treated similarly to those with IBC. This treatment consists of breast-conserving surgery (BCS) usually followed by radiotherapy or endocrine treatment for oestrogen (ER)-positive DCIS in some countries,^1^ or of mastectomy.^5^ Consequently, many women with DCIS undergo overtreatment. The development and use of models capable of effectively identifying low-risk DCIS patients would enable offering active surveillance, rather than conventional, radical treatments, to these women.

Multiple ongoing trials, the COMET, LORIS, LORETTA and LORD,^6^^,^^7^ are investigating whether active surveillance for grade I or II DCIS is safe. However, the use of grade as a clinical test is complicated by inter-observer variability^8^ and conflicting evidence regarding grade association with iIBC risk.^9^^,^^10^ Comprehensive studies have identified several risk factors in DCIS, including high human epidermal growth factor 2 (HER2),^11^ p16 expression,^10^ cyclooxygenase-2 (COX-2) expression,^11^ periductal fibrosis^11^ and clinical and radiological factors such as young age,^9^^,^^10^ positive margins,^9^^,^^10^ mode of presentation^10^ and DCIS size.^9^ A number of DCIS risk classifiers based on clinico-pathological factors^12^, ^13^, ^14^ and, more recently, biomolecular variables^15^^,^^16^ have been developed.

However, the need for decision aids for women with DCIS persists,^17^ as existing classifiers have been developed or externally validated with patient series of limited size and or representativeness. Additionally, biomolecular classifiers have not been widely used, as there are no assays yet to indicate active surveillance for patients with DCIS.

Routinely processed haematoxylin and eosin (H&E) stained whole-slide images (WSIs) of DCIS lesions provide a comprehensive view of cells and tissue, facilitating the extraction of biomarkers like grade. With the growing digitisation of pathology laboratories, there is an enticing opportunity in exploring computational, image-based features from digitised WSIs. This promise was reinforced by Klimov et al., in 2019, who developed a WSI-based DCIS risk classifier using a machine-learning model that leveraged a curated array of features derived from tissue segmentations.^18^ However, this method required pathologist annotations on a large number of samples, which is expensive and time consuming. Deep learning models can be applied to identify features predicting DCIS outcomes from WSIs without manual feature engineering. They have demonstrated capacity in: detecting DCIS,^19^ distinguishing DCIS from other breast cancer subtypes,^20^ grading DCIS,^21^ as well as stratifying patients for prognostic outcomes based on WSIs.^22^, ^23^, ^24^ However, the latter task is inherently challenging due to heterogeneity and bias within patient populations, intricate interactions among variables, and unforeseen events or confounders that defy modelling. This is further complicated by the lack of prior knowledge of predictive morphological features and the scarcity of large, high-quality datasets with sufficient follow-up.

Given the complexity of predicting recurrence outcomes in DCIS, Solin et al. have suggested integrating features from different data modalities^25^ to yield a more comprehensive patient profile. This approach was substantiated by Zhou et al. who demonstrated improved survival prediction for colon adenocarcinoma patients by integrating deep learning-based image features with clinical and genomic variables.^22^

In this study, we present deep learning models trained, in an end-to-end fashion, to predict pure DCIS recurrence as iIBC directly from WSIs on a large-scale high-quality dataset.

We demonstrate that risk stratification for DCIS can be achieved using image-only deep learning models. We further show that integrative training with predictive clinico-pathological features slightly enhanced risk stratification and merits further investigation. By accurately identifying patients with low-risk of recurrence during evaluation periods of five and 20 years in a 5-fold nested cross-validation, our models illustrate that deep-learning based stratification of DCIS lesions on H&E-stained WSIs has the potential to mitigate overtreatment of DCIS.

## Methods

### Patient selection and study design

**Fig. 1:**
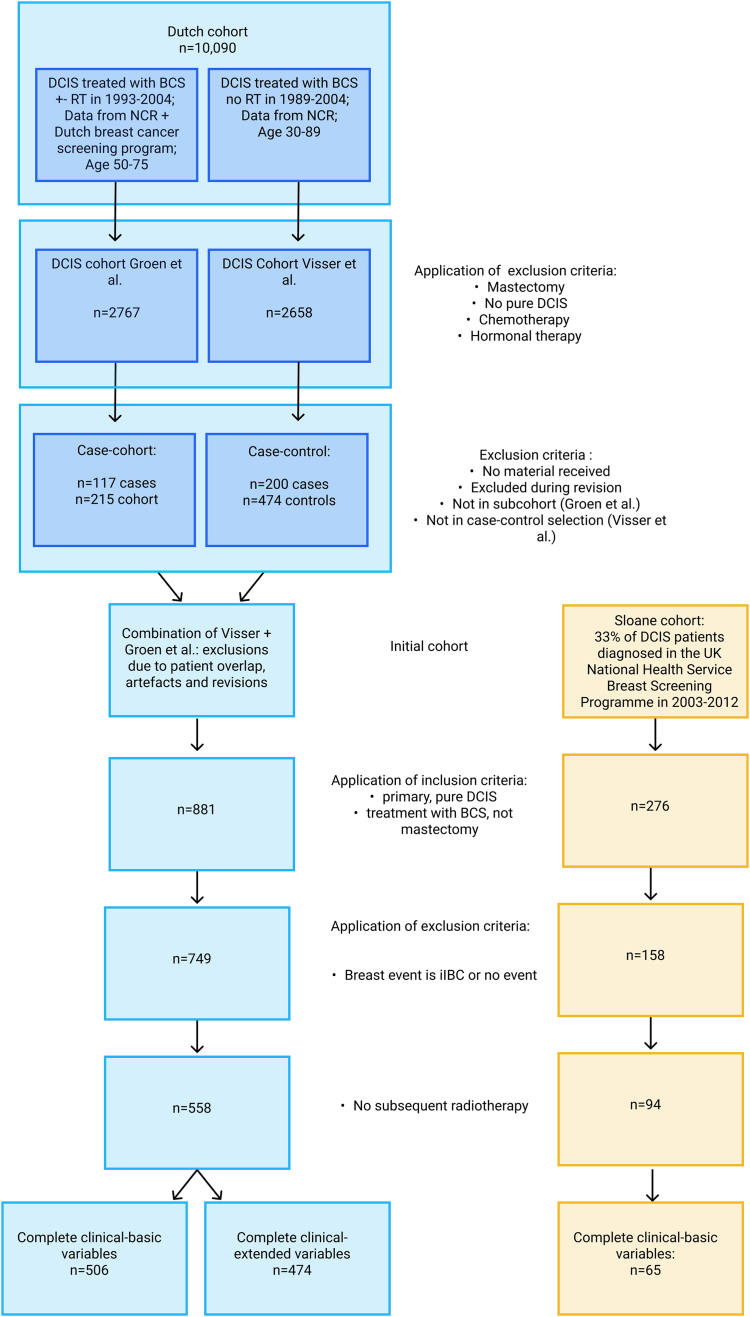
**Flowchart of patient inclusion.** Dutch dataset (left); Sloane dataset (right).

In our study, we utilised two cohorts, the Dutch and Sloane cohorts. Inclusion criteria comprised a primary, pure DCIS diagnosis, initial treatment with BCS, and recorded follow-up on breast events. Exclusion criteria limited breast events to iIBC or none, and prohibited adjuvant treatments like radiotherapy or endocrine therapy. For both cohorts, patient characteristics are presented in Table 1, and patient selection is detailed in Fig. 1.

**Table 1:** Patient characteristics of the Dutch dataset and the Sloane external test cohort.

Dataset	Dutch (n = 558)		Sloane (n = 94)	
Median follow-up	240 months		78 months	
IQR follow-up	60 months		44 months	
Patient characteristics	Low risk	High risk	Low risk	High risk
Total	343	215	72	22
Median recurrence-free time	240	74	80.5	42
IQR recurrence-free time	60	79	40.25	30
Sex-female	343	215	72	22
Age patient mean	59.7	57.25	59.43	60.45
Age patient std	10.93	11.33	6.36	6.52
Deceased	116	102		
Vital	227	113		
Age slide mean	27.2	27.99		
Age slide std	3.56	3.56		
GRADE 1	71	29	11	5
GRADE 2	198	114	33	5
GRADE 3	73	72	27	12
GRADE Missing	1	0	1	0
HER2 Negative	238	134	47	14
HER2 Positive	70	62	14	6
HER2 Borderline	4	3	3	0
HER2 Missing	31	16	8	2
ER Negative	55	37	9	4
ER Positive	260	163	61	18
ER Missing	28	15	2	0
PR Negative	113	76	17	7
PR Positive	198	122	39	7
PR Missing	32	17	16	8
COX2 1	75	22		
COX2 2	172	110		
COX2 3	52	53		
COX2 Missing	44	30		
P16 Negative	160	100		
P16 Positive	144	89		
P16 Missing	39	26		
P53 Abnormal	48	29		
P53 Normal	256	158		
P53 Missing	39	28		

The datasets, as presented, were used for the image-only models. For models incorporating clinical variables, the same datasets were used with exclusions applied to patients missing required clinical inputs. Patient characteristics for these datasets are detailed in the Supplement.

#### Dutch cohort

From a Dutch nationwide cohort of 10,090 women diagnosed between 1989 and 2005 with follow-up until 2019,^9^ slides of good quality with confirmed pure DCIS were included in the case–control series used by Visser et al.^11^ and the case-cohort series used by Groen et al.^26^ (Fig. 1). From these series, we selected 881 patients which met our inclusion criteria. After applying the exclusion criteria, the Dutch dataset comprised 558 patients, with 34% (116/343) patients without iIBC recurrence and 47% (102/215) patients with iIBC recurrence having died during the follow-up period. Each WSI was manually reviewed for the exclusive presence of DCIS, and grading was performed and revised by three expert pathologists (J. Wesseling, E.J. Groen, and K. van de Vijver). Slides were assessed on morphologic characteristics, including DCIS architecture and nuclear grade, the presence of calcifications and necrosis, and microenvironmental characteristics like stromal features and the presence of lymphocytes. Interrater variability was judged to be within expected ranges.

#### Sloane cohort

In the Sloane prospective cohort, which comprises 33% of DCIS patients diagnosed within the UK National Health Service Breast Screening Programme between 2003 and 2012,^27^ a selection of screen-detected pure DCIS cases with clear surgical margins >1 mm was made. A subset of 276 patient samples fulfilled our inclusion criteria, and after applying the exclusion criteria, the dataset was reduced to 94 patients. Information on patient mortality was not available.

### Data preparation

#### Imaging and image pre-processing

Formalin-fixed paraffin-embedded tissue sections of DCIS lesions were stained with H&E and scanned at 20× magnification, using a Leica Aperio AT2 scanner in the Dutch cohort, and a Hamamatsu Nanozoomer 2.0-HT scanner in the Sloane cohort. We then applied a U-Net segmentation model, trained in our laboratory, to the WSIs to produce tissue masks. WSIs were tiled into non-overlapping patches of 512 × 512 pixels at a resolution of 1.0 μm/pixel, providing clear views of entire medium-sized ducts and individual cells. Finally, patches with less than 30% segmented tissue were removed.

#### Construction of datasets with clinical variables

From the available clinicopathological variables, we made two selections which we refer to as “basic”, and “extended” clinical variables, based on Visser et al., 2018.^11^ The basic clinical variables included routinely assessed variables and were available in both data cohorts: age at diagnosis, DCIS grade, ER, progesterone receptor (PR), and HER2 overexpression. The extended clinical variables included p16, and COX-2 scores in addition to the basic variables. The encoding of clinical variables is shown in Table 2. Datasets were constructed for each clinical variable selection by excluding patients with missing data, as missing values were concentrated within the same patients when poor tissue quality prevented immunohistochemistry stainings. In the Dutch cohort, 506 patients had complete basic clinical variables, and 477 patients had complete extended clinical variables. In the Sloane cohort, 65 patients had complete basic clinical variables; extended clinical variables were not available.

**Table 2:** Overview of metadata availability and encoding across cohorts.

Patient Characteristics	Dataset	Encoding original	Encoding DL-Integrative Model Features	Encoding Cox-PH Model Features
Age at diagnosis	D,S	years	Ordinal: years	Ordinal: years
Histologic grade	D, S	1, 2, 3	Ordinal: 1, 2, 3	0 if 1 or 2; 1 if 3
HER2	D, S	Negative is in [0, 1+]; Borderline = 2+; Positive = 3+	0 if Negative or Borderline; 1 if Positive	0 if Negative; 1 if Positive
ER	D, S	Negative = 0–9%; Positive ≥ 10% of tumour cells stained	0 if Negative; 1 if Positive	0 if Negative; 1 if Positive
PR	D, S	Negative; Positive	0 if Negative; 1 if Positive	0 if Negative; 1 if Positive
COX2 score	D, S	1, 2, 3	Ordinal: 1, 2, 3	0 if 1; 1 if 2 or 3
p16	D	Negative if p16 score 0–100; Positive if p16 score >100	0 if Negative; 1 if Positive	0 if Negative; 1 if Positive

Clinical variables were assessed via immunohistochemistry, except for grade and age at diagnosis. In the “Dataset” column, “D” indicates availability in the Dutch dataset, and “S” indicates availability in the Sloane dataset.

DL, deep learning; Cox-PH, Cox proportional hazards.

#### Definition of time and outcome variables

We defined the dependent variable “recurrence-free time” as the number of months within the evaluation period for patients without a recurrence (“low-risk”) or the time in months until an iIBC recurrence occurred within the evaluation period for “high-risk” patients. To assess model performance within this period, the outcome variable was also adjusted accordingly. The follow-up variable refers to the time from diagnosis to the end of follow-up, regardless of whether a recurrence occurred.

### Model, training and assessment

#### Nested cross-validation

We split the Dutch dataset into five outer folds, ensuring an even distribution of covariates across splits. Within each outer split, we applied 5-fold cross-validation using GroupStratifiedKFold (scikit-learn v.1.3.2^28^), stratifying by invasive recurrence outcome and grouping by tissue block ID to ensure that all tiles from a patient’s WSI were within a single inner fold.

#### Models overview

We developed four models using deep learning or Cox proportional hazards (Cox-PH) modelling. The deep learning models comprised an image-only model and an integrative model that combined imaging data with basic clinical variables. The Cox-PH models were based on either clinical-basic or clinical-extended variables. Models were trained on an NVIDIA RTX A6000 GPU with 48 GB of memory.

#### Deep learning models

**Fig. 2:**
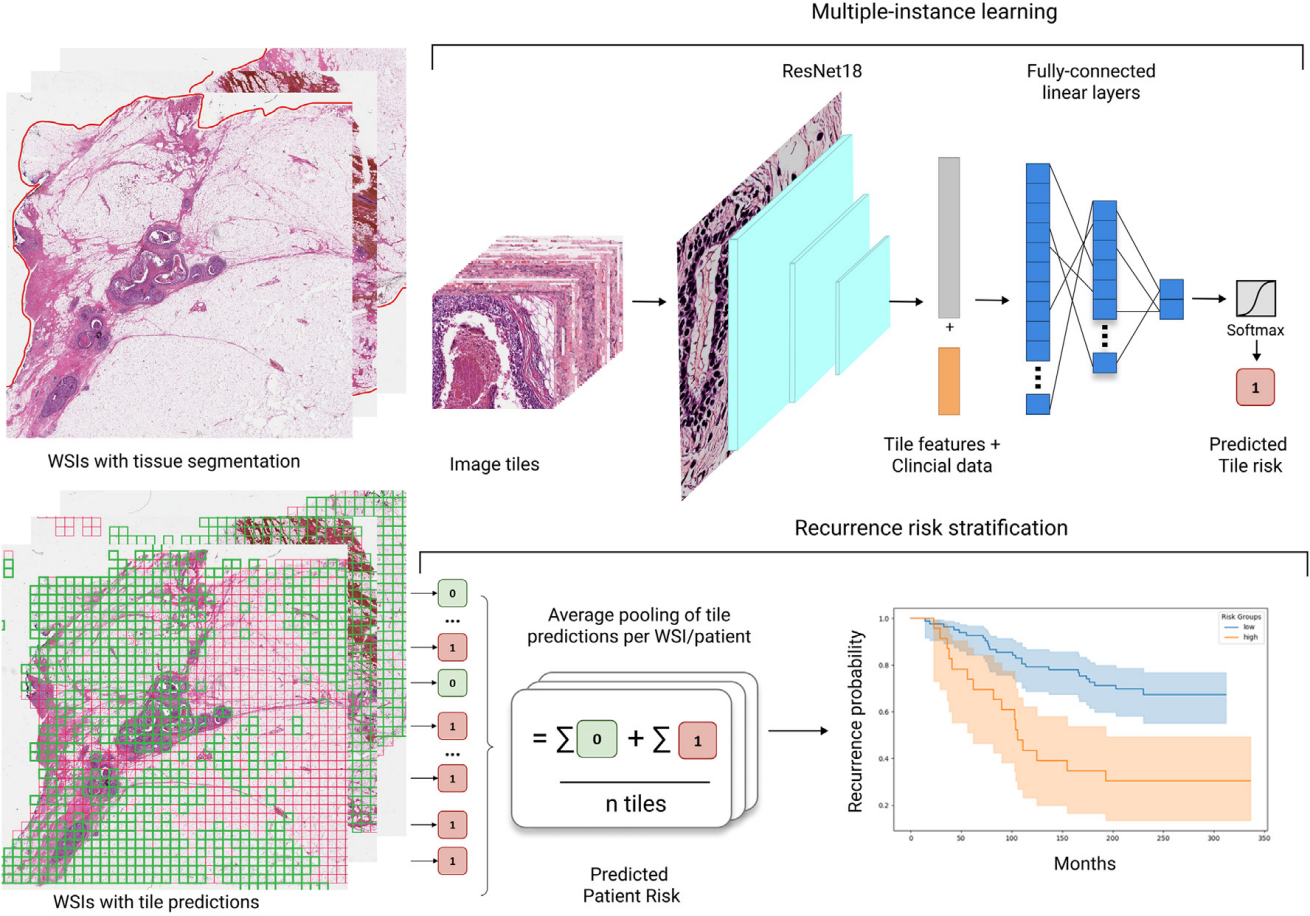
**Pipeline of the deep learning models.** Tissue areas of whole-slide images (WSIs) are segmented and tiled without overlap. Tiles are normalised using ImageNet normalisation and processed through a ResNet18 encoder pre-trained on ImageNet. In the integrative models, tile-level features are concatenated with patient-level clinical variables; otherwise, the model operates as an image-only model. These combined features are fed into a multi-layer perceptron to predict patient risk, with end-to-end training occurring using multi-instance learning.

Our models were based on tile-supervision multiple instance learning (TS-MIL), using weak labelling in which each tile was labeled with the corresponding patient outcome. Fig. 2 shows the TS-MIL model, comprising an encoder, consisting of the first three blocks of a pretrained ResNet18, paired with a two-layer MLP serving as a decoder. We found that a small network was less prone to overfitting. We pre-trained the ResNet18 using contrastive self-supervised learning on the TCGA-breast cancer dataset^28^ using MocoV2^29^ implemented in the vissl framework (v.0.1.6),^30^ see^31^ for further details. In integrative models, following the forward pass of image patches through the encoder, the tile embeddings were concatenated with the corresponding patient’s clinical features, enabling end-to-end training.

Model training and evaluation were conducted using the PyTorch Lightning library (v. 1.6.4). After conducting a hyperparameter search, we employed the Adam optimizer with an initial learning rate of 3e-5 and a weight decay of 5e-4. A batch size of 512 was used, and models were trained using the binarised outcome variable. A custom batch sampler ensured equal sampling of patches from each WSI in every training epoch. ImageNet normalization was applied to patches, and horizontal and vertical flips were used as data augmentations. More advanced data augmentation techniques^32^^,^^33^ did not improve validation performance. To address potential overfitting, we implemented a step-wise learning rate reduction, decreasing the learning rate by a factor of 2 at every epoch. The focal loss function was used, utilizing only the gamma factor set to its default value of 2, increasing the contribution of hard examples to the loss relative to easy ones. Models were evaluated five times per epoch to identify the optimal checkpoint, with training limited to five epochs to reduce the risk of overfitting. For each fold of the outer cross-validation, an ensemble model was constructed using the best-performing checkpoints from the corresponding inner cross-validation folds. The optimal checkpoints were selected based on the area under the receiver operating characteristic curve (AUC), calculated using the ‘torchmetrics` library (v0.11.2).^34^ The binarization threshold for each ensemble model was defined as the average of the optimized thresholds derived from the individual models within the ensemble.

#### Cox proportional hazards models

We implemented Cox-PH models to predict time-to-recurrence using lifelines package v.0.27.8^35^ using the datasets constructed for clinical variable selections. Proportional hazards and linearity for quantitative predictors, as required by Cox regression assumptions, were validated. The start and origin times were both defined as the date of diagnosis (recorded by month), and the end time was the recurrence-free time variable. Deceased patients were not censored, as the date of death was unavailable; they were considered at risk until the end of the recorded follow-up. However, the proportion of IBC-caused deaths can be assumed to be low with approximately 2.9%.^36^ In our hyperparameter search, we used the hyperparameters alpha = 0.05, l1_ratio = [0.0, 0.1] and penaliser = [0.0, 0.1]. The final Cox-PH models were trained with the whole training data in each outer split.

### Statistics

We assessed model performance with the AUC, next to the negative predictive value (NPV), sensitivity, and specificity calculated based on binarised risk predictions. For risk binarisation, model-specific thresholds optimised for maximum accuracy were used. Metrics were calculated using the scikit-learn (v. 1.3.2) package.^28^

To compare the predicted high-risk and low-risk groups, we obtained the hazard ratio (HR) and its associated p-value by fitting Cox-PH models. Additionally, we calculated the recurrence rate and the percentage of censored cases within each predicted risk group, and compared them using a two-tailed t-test. We assessed statistical significance in the comparison of predicted risk groups with a p-value threshold of 0.05 and the s-value, which indicates the number of hypothetical events (e.g., only heads in a series of coin tosses) needed to achieve the corresponding p-value.^37^^,^^38^ We generated Kaplan–Meier (KM) curves using the KaplanMeierEstimator from the lifelines package (v.0.27.8) to visually interpret the predicted risk groups, with statistical significance evaluated using the log-rank test. In the KM curves, we also display mortality within each predicted risk group, detailing both total deaths and deaths among patients at risk.

To evaluate outer cross-validation models on the internal dataset, we pooled test predictions from each split, and calculated performance metrics with 95% confidence intervals (CIs). For the external dataset, we averaged performance across splits, and pooled hazard ratios and 95% CIs using mean inverse-variance weighting. Finally, we applied Hommel’s method for multiple testing corrections for p-values from across models within the same time period using stats.multitest from the statsmodels package (v.0.14.2). We evaluated models over two time periods: five years and 20 years.

### Ethics

This retrospective study was approved by the review boards of the NKI-AVL (IRBd22-110), the Netherlands Cancer Registry (request K12.281; January 3, 2013) and PALGA (LZV990; April 16, 2013).

Secondary data usage adhered to Dutch regulations and the Federa-COREON Code of Conduct under an opt-out regime, and therefore the Institutional Review Board provided a waiver for informed consent. Ethics Committee approval was granted for the Sloane DCIS study (19/LO/0648).

### Role of funders

The funders of the study had no role in study design, data collection, analysis, interpretation, or writing of the report.

## Results

### WSIs alone can stratify women with DCIS by invasive recurrence risk over a 20-year period

We first examined whether deep learning models could predict iIBC recurrence in women with DCIS from WSIs alone over a 20-year period, corresponding to median follow-up of the Dutch dataset.

**Fig. 3:**
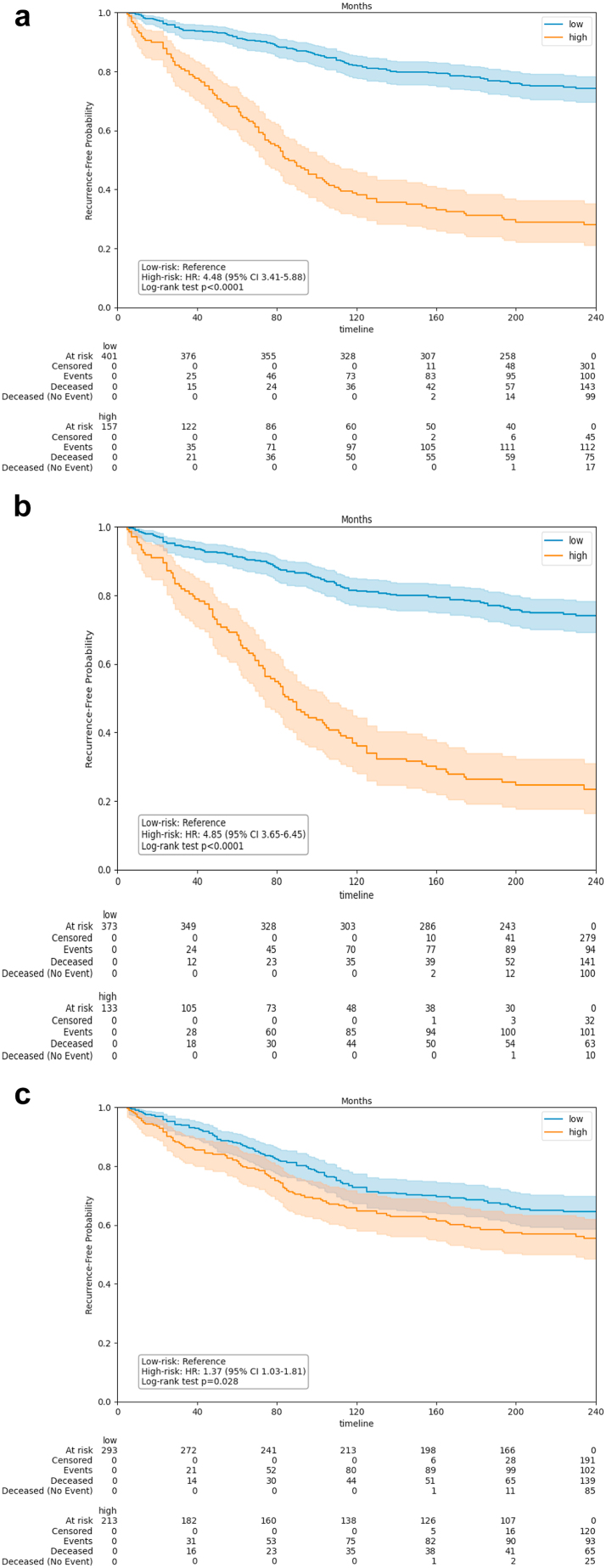
**Kaplan–Meier curves for models evaluated on 20-year follow-up in the Dutch test cohort.** (a) Image-only model, (b) Integrative model, (c) Clinical-basic Cox-PH model. The curves are based on combined predictions from models in outer cross-validation. The shaded area represents 95% confidence intervals. Abbreviations: low: predicted low-risk group; high: predicted high-risk group; HR: hazard ratio.

Using the pooled test results from a nested cross-validation on this dataset, image-only models achieved an AUC of 0.75 (95% CI: 0.70–0.79) and an NPV of 0.79 (95% CI: 0.74–0.83) (Table 3). For risk stratification, the models’ predictions were most aligned with hazard ratios ranging from 3.41 to 5.88 (HR = 4.48, p < 0.0001 [Wald-test, multiple testing corrected using Hommel’s method]), as shown in Fig. 3.

**Table 3:** Performance metrics of recurrence prediction models evaluated on the Dutch dataset.

Model	Follow-up (years)	ROC-AUC	NPV	Specificity	Sensitivity	Hazards ratio	HR p-value	HR s-value
Cox-PH-clinical-basic	5	0.57 (0.5, 0.63)	0.86 (0.83, 0.9)	0.72 (0.68, 0.76)	0.35 (0.24, 0.45)	1.57 (1.0, 2.47)	0.059	4.1
DL-image-only	5	0.71 (0.65, 0.77)	0.92 (0.89, 0.95)	0.71 (0.67, 0.75)	0.65 (0.55, 0.75)	4.3 (2.79, 6.61)	<0.0001	32.86
DL-integrative	5	0.71 (0.65, 0.78)	0.92 (0.89, 0.95)	0.72 (0.68, 0.76)	0.63 (0.52, 0.74)	4.04 (2.56, 6.38)	<0.0001	27.3
Cox-PH-clinical-basic	20	0.57 (0.52, 0.62)	0.64 (0.59, 0.69)	0.74 (0.69, 0.79)	0.33 (0.27, 0.4)	1.37 (1.03, 1.81)	0.041	4.6
DL-image-only	20	0.75 (0.7, 0.79)	0.79 (0.74, 0.83)	0.83 (0.79, 0.87)	0.63 (0.57, 0.7)	4.48 (3.41, 5.88)	<0.0001	86.1
DL-integrative	20	0.75 (0.7, 0.79)	0.77 (0.73, 0.82)	0.84 (0.8, 0.88)	0.61 (0.54, 0.67)	4.85 (3.65, 6.45)	<0.0001	86.95

Results are based on combined predictions from an outer cross-validation and assessed over 5-year and 20-year (median) follow-up periods.

NPV, negative predictive value; ROC-AUC, area under the receiver operating characteristic curve; HR, hazard ratio.

As a baseline comparison, we trained Cox-PH models with clinico-pathological variables on the same task. Clinical-basic models achieved an AUC of 0.57 (95% CI: 0.52–0.62) and a NPV of 0.64 (95% CI: 0.59–0.69), with a HR of 1.37 (95% CI: 1.03–1.81, p = 0.041 [Wald-test, multiple testing corrected using Hommel’s method]). Extending the selection of clinical variables did not improve these results (Supplementary Table S4).

We then evaluated whether integrating basic clinical variables into the deep learning model would improve risk stratification. The integrative models yielded an AUC of 0.75 (95% CI: 0.70–0.79) and an NPV of 0.77 (95% CI: 0.73–0.82), similar to the image-only models. Risk stratification was slightly improved, with a HR of 4.85 (95% CI: 3.65–6.45; p < 0.0001 [Wald-test, multiple testing corrected using Hommel’s method]).

Recurrence occurred in 71% of high-risk and 25% of low-risk patients with image-only models (p < 0.0001 [t-test, multiple testing corrected using Hommel’s method]), 76% and 25% with integrative models (p < 0.0001 [t-test, multiple testing corrected using Hommel’s method]), and 43% and 35% with clinical-basic models (p = 0.066 [t-test, multiple testing corrected using Hommel’s method]). Censoring rates for predicted low-risk patients ranged from 65 to 75%, while high-risk patients had rates from 24 to 56%, depending on the dataset used (Supplementary Table S5).

### Deep learning models accurately identify DCIS patients with low risk on invasive progression in clinically relevant 5-year period

We evaluated our models’ ability to accurately identify low-risk patients over a 5-year period (Table 3), as early-recurrent DCIS cases may have higher clinical relevance for intervention. The NPV for low-risk patients was 0.92 (95% CI: 0.89–0.95) for both image-only and integrative models. Specificity, reflecting the retrieval of low-risk patients, was 0.71 (95% CI: 0.67–0.75) for image-only models and 0.72 (95% CI: 0.68–0.76) for integrative models.

Image-only models achieved an AUC of 0.71 (95% CI: 0.65–0.77) and a HR of 4.3 (95% CI: 2.79–6.61, p < 0.0001 [Wald-test, multiple testing corrected using Hommel’s method]), while integrative models showed similar performance with a AUC of 0.71 (95% CI: 0.65–0.78) and a HR of 4.04 (95% CI: 2.56–6.38, p < 0.0001 [Wald-test, multiple testing corrected using Hommel’s method]). Clinical-basic models had limited performance, with an AUC of 0.57 (95% CI: 0.50–0.63), NPV of 0.86 (95% CI: 0.83–0.90), specificity of 0.72 (95% CI: 0.68–0.76), and a HR of 1.57 (95% CI: 1.00–2.47).

Supplementary Table S5 shows that both the image-only and integrative models predicted a recurrence rate of 9% for low-risk patients and 32% for high-risk patients (p < 0.0001 [t-test, multiple testing corrected using Hommel’s method]). In contrast, the clinical-basic model predicted 12% recurrence for low-risk patients and 18% for high-risk patients. Censoring rates ranged from 88% to 91% for low-risk patients and 68–82% for high-risk patients, depending on the dataset used by each model.

### External validation

We conducted validation of our models in an independent dataset from the Sloane project. Table 1 shows that compared to the Dutch dataset, this dataset exhibited a short median follow-up (78 months vs. 240 months in the Sloane vs. Dutch dataset), a lower proportion of high-risk patients (23.4% vs. 38.5% in the Sloane vs. Dutch dataset) as well as fewer HER2+ patients within the high-risk group (42.8% vs. 27.3% in the Sloane vs. Dutch dataset). In a 5-year evaluation period, clinical-basic models did not display significant risk stratification (HR = 1.23, 95% CI: 0.62–2.44, p = 0.56) (Supplementary Fig. S3 and Table S6), with an average censoring rate of 86% for predicted low-risk patients and 82% for predicted high-risk patients (Supplementary Table S7). Image-based models exhibited a high propensity to classify patients as high-risk, which prevented external validation and computation of meaningful metrics.

### Feature importance

Feature importance in clinical models centred on partial hazard ratios (pHRs) derived from Cox-PH models (Supplementary Table S3), which represent the partial effect of each covariate, adjusted for the presence of other covariates in the model. Higher grade, HER2, and COX-2 expression emerged as risk-increasing factors, while the predicted risk was reduced by higher age at diagnosis. The pHRs for ER, p16 and PR indicated a negligible effect on the models’ risk predictions. Additionally, in the clinical-extended model the pHR for ER indicated a small risk-increasing effect. Regarding image-only models, we visualised the predicted class for each tile on WSIs of high-risk and low-risk patients (Supplementary Fig. S2, A + B). We found that adjacent tissue areas exhibited similar classifications, but we did not discern patterns or tissue morphological structures that were consistently predicted with the same class label.

## Discussion

Our study, conducted on a large Dutch national cohort, demonstrates the potential of predicting the risk of developing IBC in the same (‘ipsilateral’) breast following a diagnosis of primary, pure DCIS. This risk is predicted by deep learning models based on either using WSIs alone or in conjunction with clinico-pathological features. We evaluated the classification models using nested cross validation across the median follow-up of 20 years for a complete risk assessment, and a prediction period of five years which focuses on patients with high risk of invasive progression and facilitates decision-making for active surveillance. We found that an image-based model can accurately predict the risk of invasive recurrence, demonstrating its potential for seamless integration into any hospital with a digital pathology workflow. However, successful large-scale external validation is needed before clinical implementation. Model development with a focus on explainability, along with deeper investigation into predictive image-based features, could further enhance trust in the use of deep learning models for DCIS treatment planning.

We found that image-only deep learning models effectively stratified iIBC recurrence risk when evaluated over the 20-year period (HR = 4.48, p < 0.0001 [Wald-test, multiple testing corrected using Hommel’s method]), as well as the 5-year period (HR = 4.3, p < 0.0001 [Wald-test, multiple testing corrected using Hommel’s method]). In 2019, the only existing WSI-based pure DCIS risk classifier was reported to achieve significant results (HR = 6.39, 95% CI = 3.0–13.8, p < 0.0001 [Wald test]) in a 10-year prediction period,^18^ with external validation still pending. There are relevant differences between the aforementioned study by Klimov et al. and ours. We defined high-risk events as invasive recurrences, whereas they included both *in-situ* and invasive recurrences. Our dataset was stratified on outcome achieving approximately 40% recurrences and reflecting the Dutch population's grade distribution, with 25% grade III cases, while Klimov’s independent test set consisted solely of grade III patients, with only 14.1% recurrences. In scenarios with fewer events, chance's impact on risk stratification is amplified, underscoring the need for performance measures that do not depend on a single cutoff, like the AUC, for reliable assessment. Additionally, Ghose et al. conducted a proof-of-concept study in 2023, predicting breast events within three years after DCIS using deep learning. They reported an AUC of 0.84 on an external dataset of 66 patients.^39^ Their study did not exclude patients with an invasive component in the DCIS or those who received treatments other than BCS, such as mastectomy, which can significantly alter a patient's risk profile.

In our study, we found that adding clinical variables to image-features showed a similar performance at the individual level, as seen in respective AUC and NPV values. The integrative models showed a slightly improved overall risk stratification in the 20-year evaluation period as demonstrated by the HR. Clinical data may help stabilise predictions for more uncertain cases, as seen in the external validation, where the integrative model predicted some patients as high-risk, whereas the image-only model predicted all as low-risk (Supplementary Table S6).

Cox-PH models using clinical data only achieved borderline significant risk stratification in a 20-year follow-up period. Unlike Cox-PH models, deep learning models allow capturing non-proportional or temporal effects in clinico-pathological features or histological patterns. However, Cox-PH models provide interpretability through partial hazard ratios: Our clinical models aligned with existing literature, with higher DCIS grade, COX-2 score, and HER2 expression contributing to high-risk classification, while younger age indicated lower risk.^10^^,^^11^ In a meta-analysis by Visser et al. ER and PR positivity did not show significant associations with invasive DCIS recurrence. In our clinical basic models, PR and ER showed modest effects. ER positivity weakly contributed to high-risk predictions in the clinical-extended models. While ER positivity is associated with markers of less aggressive DCIS lesions,^40^ there is variability in the evidence for the protective effect of ER-positivity on iIBC recurrence.^11^^,^^40^^,^^41^

Previous clinical classifiers^12^, ^13^, ^14^^,^^42^ have reported moderate performance and were limited by small development cohorts and insufficient external validation, and frequently omitted reporting robust performance measures like the AUC/C-index. Additionally, the classifiers were developed to identify patients who benefit from radiotherapy rather than a true ‘low risk’ population in which surgery may safely be omitted.^17^ By incorporating a comprehensive selection of risk factors with high correlations with iIBC, our clinical models suggest that such variables alone are insufficient for reliable risk stratification. Additionally, RNAseq-based classifiers^15^^,^^16^ have shown effectiveness in external validations assessing radiotherapy benefit for patients with BCS-treated DCIS, suggesting that adding this data type to our integrative model could be beneficial. However, bulk genomic data from DCIS may be diluted with normal tissue, and therefore using only histological images may be more clinically relevant.

We aimed to externally validate our models using the Sloane dataset, covering a more recent period (2003–2012) compared to the Dutch cohort (1989–2004), and accordingly had a shorter median follow-up of 78 months. Due to increased administration of radiotherapy in the last 20 years,^5^ the dataset was small (n = 94), with fewer high-risk patients (23%) compared to the Dutch dataset (39%), limiting the power to detect significant effects. Clinical-basic models showed a non-significant signal in the external dataset, likely aided by the standardisation in features’ registration, staining and scoring, while deep learning image features did not effectively transfer to the external dataset. Variations in staining, storage, and scanning practices across laboratories pose challenges for model generalisation in digital pathology. These challenges were exacerbated by compromised slide quality in the Sloane series, characterised by background noise, varying stain intensities, artefacts, and pen markings. They could not be overcome with normalisation, or augmentation and regularisation techniques.

Our study has several limitations. While evidence indicates that clinical variables are insufficient for accurately predicting long-term DCIS outcomes, our study was biased through the available selection of clinical predictors, as well as exclusions of patients without complete clinical variables. Our study used survival analysis, which benefits from easily interpreted hazard ratios. However, these have an inherent selection bias due to potential changes in patient risk over time and censoring effects. Due to the missing date of death in our data, we did not account for the time of death in our analysis. While non-IBC related death likely led to labelling of potential high-risk DCIS patients as low-risk, this also reflects real-world scenarios where a potential high-risk patient, who dies before recurrence occurs, effectively resembles a low-risk patient. The tile-supervision approach used in our deep-learning models offers limited interpretability and may overfit localised features. Despite efforts, an attention-based whole-slide supervision approach^43^^,^^44^ could not successfully be trained, unlike in a similar study predicting survival of patients with colorectal cancer,^23^ highlighting the difficulty of the task at hand.

While we had access to a national, multi-centre dataset larger than those previously used for long-term DCIS recurrence risk prediction, its size may have constrained model validation. Acquiring another large-scale, high-quality dataset akin to the Dutch dataset for external validation would be invaluable, but this is hindered by widespread radiotherapy administration and the requirement of extended follow-up. International scientific collaboration, through initiatives like Cancer Grand Challenges, may aid in overcoming these hurdles.

Robustly predicting long-term DCIS recurrence remains a challenging task, as our study demonstrates. Future research should focus on transferability of image features and directing models towards interpretable features that are verifiable in large-scale external datasets, or simplifying data complexity by leveraging biologically relevant patterns, for instance, through tissue graph or prototype modelling. In conclusion, this study demonstrates the feasibility of predicting primary, pure DCIS recurrence as ipsilateral IBC from routinely processed WSIs directly using deep learning, and indicates that multi-modal learning through integration with clinical variables may enhance risk stratification and merits further investigation. Efforts need to be made to collect large datasets that encompass clinical, imaging, and biomolecular data. This could allow the development of robust deep learning models that model disease across diverse settings. Evaluating such models in active surveillance trials could help to establish their utility and robustness. With successful validation, deep-learning DCIS recurrence models could enhance clinical practice without disrupting clinical workflow by aiding in decision-making and development of personalised DCIS management plans, thereby reducing overtreatment for women with DCIS.

### Plain language summary

Ductal carcinoma *in situ* (DCIS) is a common finding in breast cancer screening, yet 3 out of 4 women with DCIS will not develop invasive breast cancer in the same (‘ipsilateral’) breast (iIBC) or any symptoms. Therefore, many women are currently treated unnecessarily. Our deep learning models aim to predict the risk of iIBC recurrence after surgery in women diagnosed for the first time with pure DCIS.

These models use digitised microscope images of stained tumour tissue, the pathologist's assessment of the tumour grade, and clinical information like the patient’s age at diagnosis and results from protein marker tests.

We developed three types of models: one deep learning model using only the tumour tissue images, another deep learning model combining image data with clinical information (integrative), and a third statistical model using only clinical data to model the risk of recurrence over time as a baseline comparison. Fig. 2 provides a flow diagram showing how the deep learning models process and integrate the data.

Our dataset included 558 patients which were treated for DCIS only with surgical removal of the tumour and no additional treatments, and were monitored for a median of 20 years after diagnosis (Table 1). They are part of a large Dutch nationwide cohort of 10,090 women diagnosed between 1989 and 2005 (Fig. 1). Data preprocessing involved extracting smaller tumour-only patches from the giga-pixel microscopy images and organising clinical predictors into categories.

The models were trained and assessed using nested cross-validation (k = 5), with balanced outcomes and predictors across data splits. Over an evaluation period of 20 years, the image-only models achieved a negative predictive value (NPV) of 79%, an area under the curve (AUC) of 0.75, and a hazard ratio of 4.48. The integrative models produced similar results, with a slightly higher hazard ratio of 4.85. The clinical-only models showed a NPV of 64%, an AUC of 0.57, and a hazard ratio of 1.37. Protein markers HER2 and COX-2, along with high tumour grade and lower age at diagnosis, contributed to higher risk predictions in the clinical model. Additionally, when assessed over a clinically relevant 5-year period, the models showed high NPVs (image-only: NPV = 92%, integrative: NPV = 92%, clinical: NPV = 86%), reflecting their ability to accurately identify patients at low risk of developing iIBC. We attempted external validation using a 94-patient dataset from a multi-centre UK cohort, which was unsuccessful, likely due to low number of recurrences and poor-quality tissue images.

Currently, there are no widely used clinical prediction tools to assess recurrence risk for DCIS. Our findings highlight the potential of deep learning models applied to digitised tumour tissue images to address this gap. However, larger external datasets and dedicated methods aimed at improving model generalisability are needed, and further research should explore the value of including clinical and biomolecular variables.

With successful external validation, deep learning models could be integrated into clinical workflows to support clinicians in identifying patients suitable for active surveillance. This would reduce unnecessary treatment and facilitate more personalised care for women with DCIS.

## Contributors

Shannon Doyle: Literature research, model development, data analysis, data collection, data interpretation, figures, writing.

Esther Lips: Supervision, data collection, data interpretation, review, project administration.

Jelle Wesseling: Supervision, study design, review, funding acquisition, project administration.

Jonas Teuwen: Supervision, study design, software, supervision, review, funding acquisition, project administration.

Clara Sanchez: Supervision, review, project administration.

Eric Marcus: Supervision, data interpretation, review.

Francesco Dal Canton: Data collection, investigation, study design, review.

Timo Koostra: Investigation, review.

Maartje van Seijen: Data curation, review.

Ihssane Bouybayoune: Data collection, review.

Elinor Sawyer: Data collection, review.

Sarah Pinder: Data collection, review.

PRECISION consortium: Data collection, resources, review.

Lennart Mulder: Data curation, review.

Yat-Hee Liu: Data curation, review.

Alastair Thompson: Data collection, review.

All authors contributed to the editing of the final manuscript. All authors had full access to the data, and SD, LM, and YL directly accessed and verified the data in the study. All authors accept the final responsibility to submit for publication and take responsibility for the contents of the manuscript.

## Data sharing statement

The data utilised in this study was shared among members of the PRECISION Consortium under a unified funding initiative. Requests for access to the data may be accommodated upon reasonable inquiry directed to the corresponding author, subject to appropriate agreements.

## Code sharing

Code is open-source and available with publication at https://github.com/NKI-AI/paper-doyle2025.

## Declaration of AI and AI-assisted technologies in the writing process

During the preparation of this work the author(s) used OpenAI's tool ChatGPT in order to facilitate the writing process. After using this tool/service, the author(s) reviewed and edited the content as needed and take(s) full responsibility for the content of the publication.

## Declaration of interests

JT declares being a share owner of Ellogon.AI; consulting for ScreenPoint Medical (all active). JT received support to attend II-ON BMS conference. JT is also a member of the Dutch Cancer Foundation KWF—Smart Measurement committee, as well as Dutch organisation for scientific research—Veni Committee. All other authors declare no competing interests. The consortium was funded by Cancer Grand Challenges, an initiative from Cancer Research UK and the National Cancer Institute, under project Cancer Grand Challenge PRECISION (C38317/A24043).

## Appendix ASupplementary data

Supplementary Figs. S1–S3 and Tables S1–S7

Precision Consortium Members
