# Supplementary material for: Deep learning for predicting invasive recurrence of ductal carcinoma in situ: leveraging histopathology images and clinical features
Source: eBioMedicine. 2025 May 28;116:105750. doi: 10.1016/j.ebiom.2025.105750 (PMC12162051; doi:10.1016/j.ebiom.2025.105750)
Supplement: Supplementary Figs. S1–S3 and Tables S1–S7 [file mmc1.docx]

# **Supplement**

## Patient characteristics

### Patient inclusion

Additional Sloane Cohort Description

The Sloane cohort stems from the Sloane project which, since 2003, has collected comprehensive radiological, pathological, treatment and follow-up data for over 15,000 patients with screen-detected breast carcinoma in situ and atypical hyperplasia throughout the UK.

One patient’s slide and the endocrine therapy status of 24 patients were missing, which reduced the dataset to 251 slides. After applying the exclusion criteria for the type of BE, the dataset size was 158. After excluding patients treated with radiotherapy, the dataset was reduced to n=94. There were 11 patients in whom at least one of the clinicopathological metadata variables was missing. None of the patients had HER2 borderline status. The final dataset size was 83. Additionally, HER2 status was determined through in-situ hybridization rather than immunohistochemistry, as was the case for Dutch Cohort.

### Clinical variable selection

In this study, clinical variables were selected based on Visser et al. (2018), which identified biomarkers associated with IBC recurrence risk in DCIS. We included grade as a histopathological variable, as it is the diagnostic gold standard, and age at diagnosis as a clinical variable. From IHC markers, we included ER, PR, HER2, COX-2, and p16. The “basic” clinical variable set—ER, HER2, PR, age at diagnosis, and grade—aligns with the Sloane series and is widely used. The “extended” set includes p16 and COX-2 in addition to the basic variables.

### Patient Characteristics Tables

| **Dataset** | **Dutch (n=506)** | | **Sloane (n=65)** | |
| --- | --- | --- | --- | --- |
| Median Follow-up | 252 months | | 79 months | |
| IQR Follow-up | 60 months | | 39 months | |
| Patient Characteristics | Low risk | High risk | Low risk | High risk |
| Total | 308 | 198 | 52 | 13 |
| Sex-Female | All | All | All | All |
| Median recurrence-free time | 240 | 77 | 84 | 36 |
| IQR recurrence-free time | 60 | 76.5 | 36 | 24 |
| Age in years mean | 59.78 | 57.02 | 60.06 | 62.77 |
| Age in years std | 11.21 | 11.35 | 6.24 | 6.65 |
| Deceased | 110 | 94 |  |  |
| Vital | 198 | 104 |  |  |
| Age Slide mean | 27.36 | 28.05 |  |  |
| Age Slide std | 3.54 | 3.58 |  |  |
| GRADE 1 | 65 | 27 | 6 | 3 |
| GRADE 2 | 179 | 106 | 26 | 2 |
| GRADE 3 | 64 | 65 | 20 | 8 |
| HER2 Negative | 235 | 133 | 37 | 8 |
| HER2 Positive | 70 | 62 | 12 | 5 |
| HER2 Borderline | 3 | 3 | 3 | 0 |
| ER Negative | 54 | 37 | 7 | 4 |
| ER Positive | 254 | 161 | 45 | 9 |
| PR Negative | 113 | 76 | 17 | 7 |
| PR Positive | 195 | 122 | 35 | 6 |
| COX2 1 | 71 | 22 |  |  |
| COX2 2 | 169 | 110 |  |  |
| COX2 3 | 51 | 53 |  |  |
| COX2 Missing | 17 | 13 |  |  |
| P16 Negative | 155 | 100 |  |  |
| P16 Positive | 141 | 88 |  |  |
| P16 Missing | 12 | 10 |  |  |
| P53 Abnormal | 47 | 29 |  |  |
| P53 Normal | 250 | 158 |  |  |
| P53 Missing | 11 | 11 |  |  |

**Table S1.A Patient characteristics of the Dutch dataset and Sloane external test cohort after excluding patients with missing values in clinical-basic variables. These datasets served as inputs for the clinical-basic and integrative models.**

| **Dataset** | **Dutch (n=474)** | |
| --- | --- | --- |
| Median Follow-up | 252 months | |
| IQR Follow-up | 60 months | |
| Patient Characteristics | Low risk | High risk |
| Total | 290 | 185 |
| Median recurrence-free time | 240 | 75 |
| IQR recurrence-free time | 60 | 78 |
| Sex-Female | All | All |
| Age Patient mean | 59.99 | 56.84 |
| Age Patient std | 11.07 | 11.34 |
| Deceased | 105 | 88 |
| Vital | 185 | 97 |
| Age Slide mean | 27.39 | 28.07 |
| Age Slide std | 3.51 | 3.62 |
| GRADE 1 | 62 | 26 |
| GRADE 2 | 168 | 100 |
| GRADE 3 | 60 | 59 |
| HER2 Negative | 223 | 125 |
| HER2 Positive | 65 | 58 |
| HER2 Borderline | 2 | 2 |
| ER Negative | 50 | 32 |
| ER Positive | 240 | 153 |
| PR Negative | 104 | 70 |
| PR Positive | 186 | 115 |
| COX2 1 | 71 | 22 |
| COX2 2 | 169 | 110 |
| COX2 3 | 50 | 53 |
| P16 Negative | 151 | 99 |
| P16 Positive | 139 | 86 |
| P53 Abnormal | 46 | 28 |
| P53 Normal | 244 | 156 |
| P53 Missing | 0 | 1 |

**Table S1.B Patient characteristics of the Dutch dataset after excluding patients with missing values in clinical-extended variables.** This dataset served as input for the clinical-extended models.

|  | All | | Clinical-Basic Complete | | Clinical-Extended Complete | |
| --- | --- | --- | --- | --- | --- | --- |
| Patient Characteristics | train | val | train | val | train | val |
| n patients | 446.4 (441.09, 451.71) | 111.6 (106.29, 116.91) | 404.8 (399.88, 409.72) | 101.2 (96.28, 106.12) | 380.0 (375.27, 384.73) | 95.0 (90.27, 99.73) |
| Total | 446.4 (441.09, 451.71) | 111.6 (106.29, 116.91) | 404.8 (399.88, 409.72) | 101.2 (96.28, 106.12) | 380.0 (375.27, 384.73) | 95.0 (90.27, 99.73) |
| Median recurrence-free time | 204.0 (204.0, 204.0) | 203.8 (198.08, 209.52) | 204.0 (204.0, 204.0) | 204.1 (195.1, 213.1) | 206.4 (199.74, 213.06) | 205.6 (192.65, 218.55) |
| IQR recurrence-free time | 155.2 (149.69, 160.71) | 157.9 (142.3, 173.5) | 154.6 (148.27, 160.93) | 159.1 (141.44, 176.76) | 154.65 (148.13, 161.17) | 154.15 (136.4, 171.9) |
| Median Time-to-recurrence | 74.5 (72.64, 76.36) | 73.9 (63.8, 84.0) | 76.8 (74.96, 78.64) | 78.4 (71.45, 85.35) | 75.3 (73.62, 76.98) | 78.6 (71.39, 85.81) |
| IQR Time-to-recurrence | 78.8 (73.29, 84.31) | 81.5 (39.24, 123.76) | 76.65 (69.58, 83.72) | 81.15 (38.24, 124.06) | 78.0 (70.18, 85.82) | 81.15 (36.54, 125.76) |
| Median Time-to-no-recurrence | 240.0 (240.0, 240.0) | 242.4 (229.93, 254.87) | 240.0 (240.0, 240.0) | 242.4 (229.93, 254.87) | 240.0 (240.0, 240.0) | 242.4 (229.93, 254.87) |
| IQR Time-to-no-recurrence | 60.0 (60.0, 60.0) | 61.8 (56.8, 66.8) | 60.0 (60.0, 60.0) | 60.6 (58.93, 62.27) | 60.0 (60.0, 60.0) | 58.2 (54.87, 61.53) |
| OUTCOME no recurrence | 0.61 (0.6, 0.63) | 0.61 (0.57, 0.65) | 0.61 (0.6, 0.62) | 0.61 (0.56, 0.65) | 0.61 (0.6, 0.62) | 0.61 (0.56, 0.67) |
| OUTCOME recurrence | 0.39 (0.37, 0.4) | 0.39 (0.35, 0.43) | 0.39 (0.38, 0.4) | 0.39 (0.35, 0.44) | 0.39 (0.38, 0.4) | 0.39 (0.33, 0.44) |
| Age Patient mean | 58.76 (58.5, 59.02) | 58.75 (57.69, 59.81) | 58.7 (58.46, 58.94) | 58.7 (57.69, 59.71) | 58.76 (58.47, 59.06) | 58.75 (57.55, 59.95) |
| Age Patient std | 11.14 (11.0, 11.28) | 11.15 (10.56, 11.73) | 11.33 (11.19, 11.48) | 11.34 (10.72, 11.97) | 11.27 (11.13, 11.41) | 11.27 (10.68, 11.87) |
| Deceased | 0.39 (0.37, 0.41) | 0.39 (0.31, 0.47) | 0.4 (0.38, 0.42) | 0.4 (0.33, 0.48) | 0.41 (0.39, 0.42) | 0.4 (0.33, 0.48) |
| Vital | 0.61 (0.59, 0.63) | 0.61 (0.53, 0.69) | 0.6 (0.58, 0.62) | 0.6 (0.52, 0.67) | 0.59 (0.58, 0.61) | 0.6 (0.52, 0.67) |
| Age Slide mean | 27.5 (27.39, 27.62) | 27.5 (27.02, 27.97) | 27.63 (27.52, 27.73) | 27.62 (27.19, 28.05) | 27.65 (27.55, 27.75) | 27.65 (27.26, 28.04) |
| Age Slide std | 3.57 (3.53, 3.61) | 3.57 (3.41, 3.73) | 3.57 (3.53, 3.61) | 3.57 (3.42, 3.72) | 3.57 (3.52, 3.61) | 3.57 (3.38, 3.75) |
| GRADE 1 | 0.18 (0.17, 0.18) | 0.18 (0.16, 0.2) | 0.18 (0.17, 0.19) | 0.18 (0.16, 0.21) | 0.19 (0.18, 0.19) | 0.18 (0.16, 0.21) |
| GRADE 2 | 0.56 (0.55, 0.57) | 0.56 (0.54, 0.58) | 0.56 (0.56, 0.57) | 0.56 (0.54, 0.59) | 0.56 (0.56, 0.57) | 0.57 (0.54, 0.59) |
| GRADE 3 | 0.26 (0.26, 0.27) | 0.26 (0.24, 0.28) | 0.25 (0.24, 0.27) | 0.25 (0.22, 0.29) | 0.25 (0.24, 0.26) | 0.25 (0.22, 0.28) |
| HER2 Negative | 0.67 (0.66, 0.67) | 0.67 (0.64, 0.7) | 0.73 (0.72, 0.73) | 0.73 (0.7, 0.75) | 0.73 (0.72, 0.74) | 0.73 (0.7, 0.77) |
| HER2 Positive | 0.23 (0.23, 0.24) | 0.24 (0.21, 0.26) | 0.26 (0.25, 0.27) | 0.26 (0.23, 0.29) | 0.26 (0.25, 0.27) | 0.26 (0.22, 0.29) |
| HER2 Borderline | 0.01 (0.01, 0.01) | 0.01 (0.0, 0.03) | 0.01 (0.01, 0.01) | 0.01 (-0.0, 0.03) | 0.01 (0.01, 0.01) | 0.01 (0.0, 0.01) |
| ER Negative | 0.16 (0.15, 0.18) | 0.16 (0.11, 0.22) | 0.18 (0.16, 0.2) | 0.18 (0.13, 0.23) | 0.17 (0.16, 0.19) | 0.17 (0.13, 0.21) |
| ER Positive | 0.76 (0.74, 0.77) | 0.76 (0.71, 0.8) | 0.82 (0.8, 0.84) | 0.82 (0.77, 0.87) | 0.83 (0.81, 0.84) | 0.83 (0.79, 0.87) |
| PR Negative | 0.34 (0.33, 0.34) | 0.34 (0.32, 0.36) | 0.37 (0.37, 0.38) | 0.37 (0.35, 0.4) | 0.37 (0.36, 0.37) | 0.36 (0.34, 0.39) |
| PR Positive | 0.57 (0.57, 0.58) | 0.57 (0.54, 0.61) | 0.63 (0.62, 0.63) | 0.63 (0.6, 0.65) | 0.63 (0.63, 0.64) | 0.64 (0.61, 0.66) |
| COX2 1 | 0.18 (0.16, 0.19) | 0.18 (0.12, 0.23) | 0.18 (0.17, 0.2) | 0.18 (0.13, 0.23) | 0.2 (0.18, 0.21) | 0.2 (0.14, 0.25) |
| COX2 2 | 0.51 (0.49, 0.52) | 0.5 (0.43, 0.57) | 0.55 (0.53, 0.57) | 0.55 (0.47, 0.63) | 0.59 (0.57, 0.61) | 0.59 (0.51, 0.67) |
| COX2 3 | 0.19 (0.18, 0.2) | 0.19 (0.14, 0.24) | 0.2 (0.19, 0.22) | 0.2 (0.15, 0.26) | 0.21 (0.2, 0.23) | 0.22 (0.16, 0.27) |
| P16 Negative | 0.47 (0.45, 0.48) | 0.47 (0.42, 0.52) | 0.51 (0.49, 0.52) | 0.51 (0.45, 0.56) | 0.53 (0.51, 0.54) | 0.53 (0.47, 0.58) |
| P16 Positive | 0.42 (0.4, 0.43) | 0.42 (0.37, 0.46) | 0.45 (0.44, 0.47) | 0.45 (0.4, 0.51) | 0.47 (0.46, 0.49) | 0.47 (0.42, 0.53) |
| P53 Abnormal | 0.14 (0.13, 0.15) | 0.14 (0.1, 0.18) | 0.15 (0.14, 0.16) | 0.15 (0.1, 0.2) | 0.16 (0.15, 0.16) | 0.16 (0.12, 0.2) |
| P53 Missing | 0.12 (0.11, 0.13) | 0.12 (0.1, 0.14) | 0.04 (0.04, 0.05) | 0.04 (0.03, 0.06) | 0.0 (0.0, 0.0) | 0.0 (-0.0, 0.01) |
| P53 Normal | 0.74 (0.74, 0.75) | 0.74 (0.71, 0.77) | 0.81 (0.79, 0.82) | 0.81 (0.76, 0.85) | 0.84 (0.84, 0.85) | 0.84 (0.8, 0.89) |
| P53 Missing |  |  | 0.04 | (0.03, 0.06) |  |  |
| P53 Normal |  |  | 0.81 | (0.76, 0.85) |  |  |

**Table S2. Patient characteristics for the Dutch datasets, presented as percentages in outcome and clinical variables.** Values represent averages of training and validation datasets across outer splits. Recurrence was defined as ipsilateral invasive breast cancer

## Results

### Supplementary Tables

### Additional Results Dutch Cohort

| **Model** | **ER** | **HER2** | **Grade** | **Age at diagnosis** | **PR** | **p16** | **COX-2** |
| --- | --- | --- | --- | --- | --- | --- | --- |
| Cox-Clinical | 1.04 (0.95, 1.12) | 1.28 (1.23, 1.34) | 1.37 (1.31, 1.43) | 0.99 (0.99, 0.99) | 1.02 (0.98, 1.07) | NA | NA |
| Cox-Clinical-Extended | 1.17 (1.06, 1.29) | 1.3 (1.23, 1.36) | 1.32 (1.27, 1.38) | 0.99 (0.98, 0.99) | 0.99 (0.94, 1.04) | 0.99 (0.94, 1.04) | 1.66 (1.53, 1.8) |

**Table S3. Average partial hazard ratios from Cox proportional hazards models trained on the Dutch dataset, evaluated over the full follow-up period.** Results are based on outer cross-validation, with mean values across splits presented along with 95% confidence intervals (CIs) in brackets.

| **Model** | **Follow-up (years)** | **ROC-AUC** | **NPV** | **Specificity** | **Sensitivity** | **Hazards Ratio** | **HR p-value** | **HR s-value** |
| --- | --- | --- | --- | --- | --- | --- | --- | --- |
| Cox-Clinical-Extended | 5 | 0.57 (0.51, 0.64) | 0.86 (0.82, 0.89) | 0.77 (0.73, 0.81) | 0.28 (0.17, 0.38) | 1.56 (0.98, 2.48) | 0.059 | 4.1 |
| Cox-Clinical-Extended | 20 | 0.59 (0.54, 0.64) | 0.65 (0.6, 0.69) | 0.8 (0.75, 0.84) | 0.3 (0.23, 0.36) | 1.36 (1.01, 1.82) | 0.041 | 4.6 |

**Table S5 . Performance metrics of the clinical-extended model evaluated on the Dutch test dataset.** Results are based on combined predictions from an 4uter cross-validation and assessed over 5-year and 20-year (median) follow-up periods. Abbreviations: NPV, negative predictive value; ROC-AUC, area under the receiver operating characteristic curve; HR, hazard ratio.

| **Model** | **Follow-up (years)** | **# LR-P** | **# HR-P** | **# Misclassified LR-P** | **# Misclassified HR-P** | **% Recurrence in LR-P** | **% Recurrence in HR-P** | **% Recurrence**  **p-value** | **% Recurrence s-value** | **% Censored LR-P** | **% Censored HR-P** |
| --- | --- | --- | --- | --- | --- | --- | --- | --- | --- | --- | --- |
| Cox-Clinical | 5 | 293 | 213 | 36 | 174 | 0.12 | 0.18 | 0.062 | 4.01 | 0.88 | 0.82 |
| Cox-Clinical-Extended | 5 | 291 | 184 | 37 | 149 | 0.13 | 0.19 | 0.062 | 4.01 | 0.87 | 0.81 |
| DL-Image-only | 5 | 401 | 157 | 35 | 106 | 0.09 | 0.32 | <0.0001 | 36.32 | 0.91 | 0.68 |
| DL-Integrative | 5 | 373 | 133 | 33 | 91 | 0.09 | 0.32 | <0.0001 | 30.32 | 0.91 | 0.68 |
| Cox-Clinical | 20 | 293 | 213 | 102 | 120 | 0.35 | 0.44 | 0.066 | 3.92 | 0.65 | 0.56 |
| Cox-Clinical-Extended | 20 | 291 | 184 | 102 | 104 | 0.35 | 0.43 | 0.066 | 3.92 | 0.65 | 0.57 |
| DL-Image-only | 20 | 401 | 157 | 100 | 45 | 0.25 | 0.71 | <0.0001 | 76.34 | 0.75 | 0.29 |
| DL-Integrative | 20 | 373 | 133 | 94 | 32 | 0.25 | 0.76 | <0.0001 | 78.41 | 0.75 | 0.24 |

**Table S5 Predicted risk groups in the Dutch test dataset evaluated over 5-year and 20-year (median) follow-up periods: Misclassification, Recurrence, and Censoring Outcomes.** Results are based on combined predictions from an outer cross-validation. The number (#) of predicted high-risk and low-risk patients is independent of the evaluation period. Abbreviations: HR-P: predicted high-risk patients with invasive recurrence; LR-P: predicted low-risk patients without invasive recurrence.

### Results - Sloane cohort (External validation)

| **Model** | **Follow-up (years)** | **ROC-AUC** | **NPV** | **Specificity** | **Sensitivity** | **Hazards Ratio** | **HR p-value** | **HR s-value** |
| --- | --- | --- | --- | --- | --- | --- | --- | --- |
| Cox-Clinical | 5 | 0.52 (0.51, 0.54) | 0.85 (0.84, 0.86) | 0.78 (0.75, 0.82) | 0.28 (0.21, 0.35) | 1.23 (0.62, 2.44) | 0.56 | 0.83 |
| DL-Image-only | 5 | 0.35 (0.33, 0.36) | NaN (NaN, NaN) | 0.0 (0.0, 0.0) | 1.0 (1.0, 1.0) | NaN (NaN, NaN) | NaN | NaN |
| DL-Integrative | 5 | 0.47 (0.42, 0.52) | NaN (NaN, NaN) | 0.18 (-0.12, 0.47) | 0.82 (0.51, 1.13) | NaN (NaN, NaN) | NaN | NaN |

**Table S6. Performance metrics for recurrence prediction models evaluated in the Sloane test dataset over a 5-year follow-up period.** Results are based on outer cross-validation, with mean values presented along with 95% confidence intervals (CIs) in brackets. NaNs in the table indicate missing predictions for both classes. Abbreviations: NPV: Negative predictive value; AUC: Area under the receiver operating characteristic curve; HR: Hazard ratio.

| **Model** | **Follow-up (years)** | **# LR-P** | **# HR-P** | **# Misclassified LR-P** | **# Misclassified HR-P** | **% Recurrence in LR-P** | **% Recurrence in HR-P** | **% Recurrence p-value** | **% Recurrence s-value** | **% Censored LR-P** | **% Censored HR-P** |
| --- | --- | --- | --- | --- | --- | --- | --- | --- | --- | --- | --- |
| Cox-Clinical | 5 | 46.4 (45.07, 47.73) | 18.6 (17.27, 19.93) | 6.6 (6.12, 7.08) | 15.2 (14.06, 16.34) | 0.14 (0.13, 0.15) | 0.18 (0.16, 0.2) | 0.94 | 0.09 | 0.86 (0.85, 0.87) | 0.82 (0.8, 0.84) |
| DL-Image-only | 5 | 0.0 (0.0, 0.0) | 94.0 (94.0, 94.0) | 0.0 (0.0, 0.0) | 76.0 (76.0, 76.0) | NaN (NaN, NaN) | 0.19 (0.19, 0.19) | NaN | NaN | NaN (NaN, NaN) | 0.81 (0.81, 0.81) |
| DL-Integrative | 5 | 11.6 (-7.83, 31.03) | 53.4 (33.97, 72.83) | 1.8 (-1.26, 4.86) | 45.2 (28.83, 61.57) | NaN (NaN, NaN) | 0.15 (0.14, 0.16) | NaN | NaN | NaN (NaN, NaN) | 0.85 (0.84, 0.86) |

**Table S7. Predicted risk groups in the Sloane test dataset evaluated over a 5-year follow-up period: Misclassification, Recurrence, and Censoring Outcomes.** Results are based on outer cross-validation, with mean values across splits presented along with 95% confidence intervals (CIs) in brackets. Abbreviations: HR-P: predicted high-risk patients with invasive recurrence; LR-P: predicted low-risk patients without invasive recurrence. The number (#) of predicted high-risk and low-risk patients is independent of the evaluation period.

# Supplementary Figures


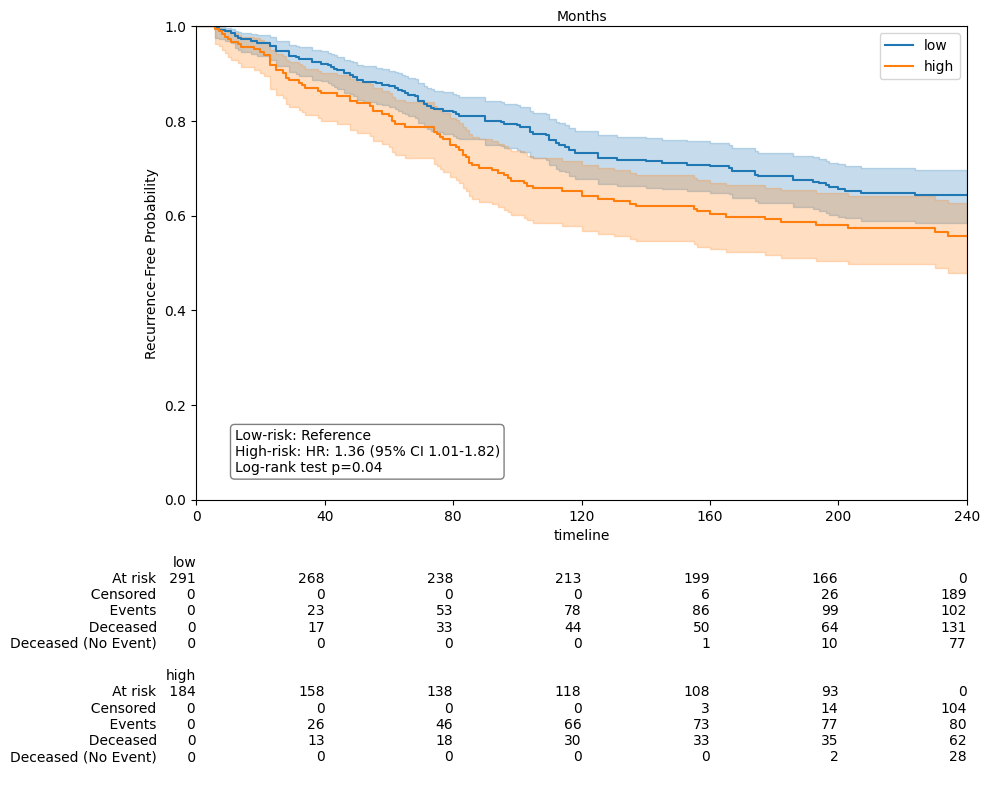


**Figure S1. Kaplan-Meier curves for clinical-extended models evaluated on 20-year follow-up in the Dutch test cohort.** The curve is based on combined predictions from models in outer cross-validation. The shaded area represents 95% confidence intervals. Abbreviations: low: predicted low-risk group; high: predicted high-risk group; HR: hazard ratio.


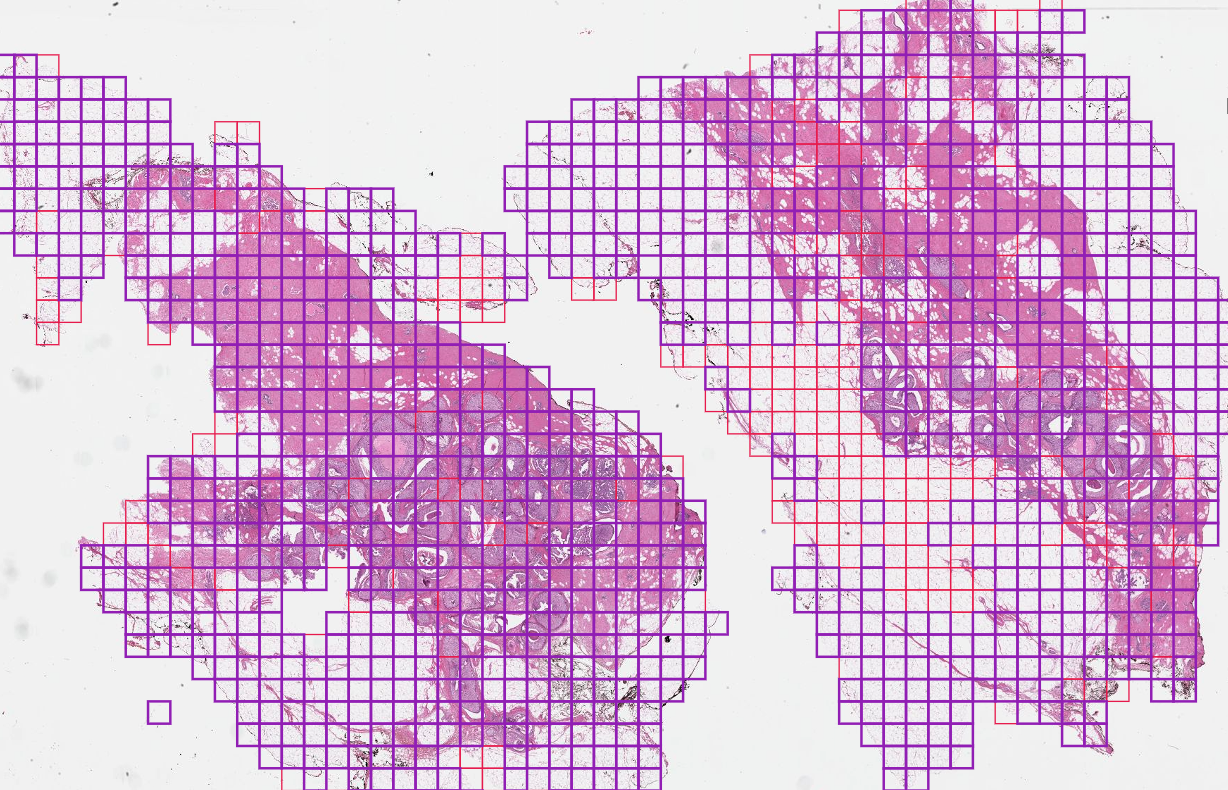


**Figure S2.A Visualisation of correctly and incorrectly predicted tiles in a WSI of a patient with recurrence.** Tiles indicated as purple are true positive predictions, and red tiles are false negative predictions.
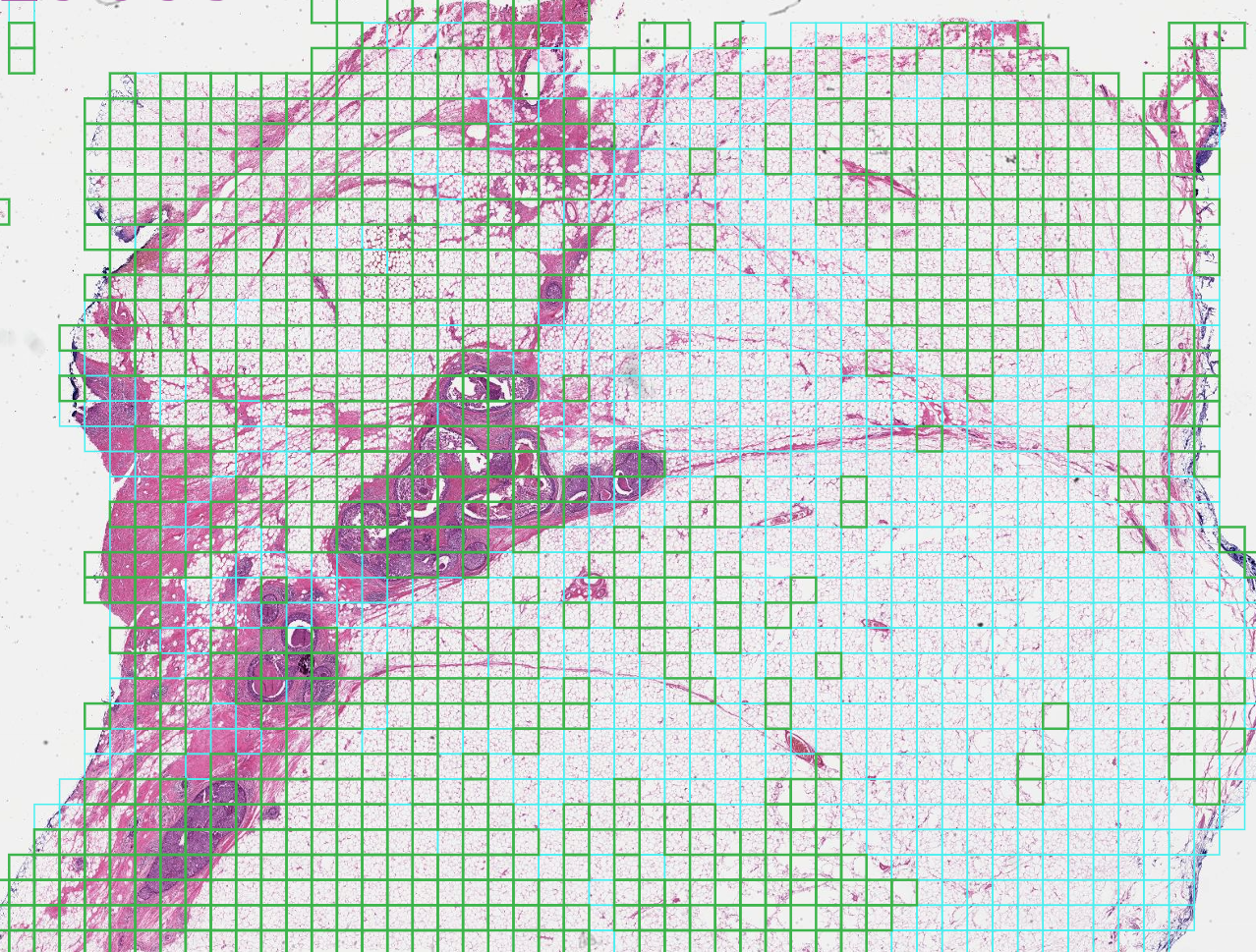


**Figure S2.B Visualisation of correctly and incorrectly predicted tiles in a WSI of a patient without recurrence.** Tiles indicated as green are true negative predictions, and blue tiles are false positive predictions.

### Results - Sloane cohort (External validation)

| **a**  Split 0 | **f**  Split 0 |
| --- | --- |
| NaN | **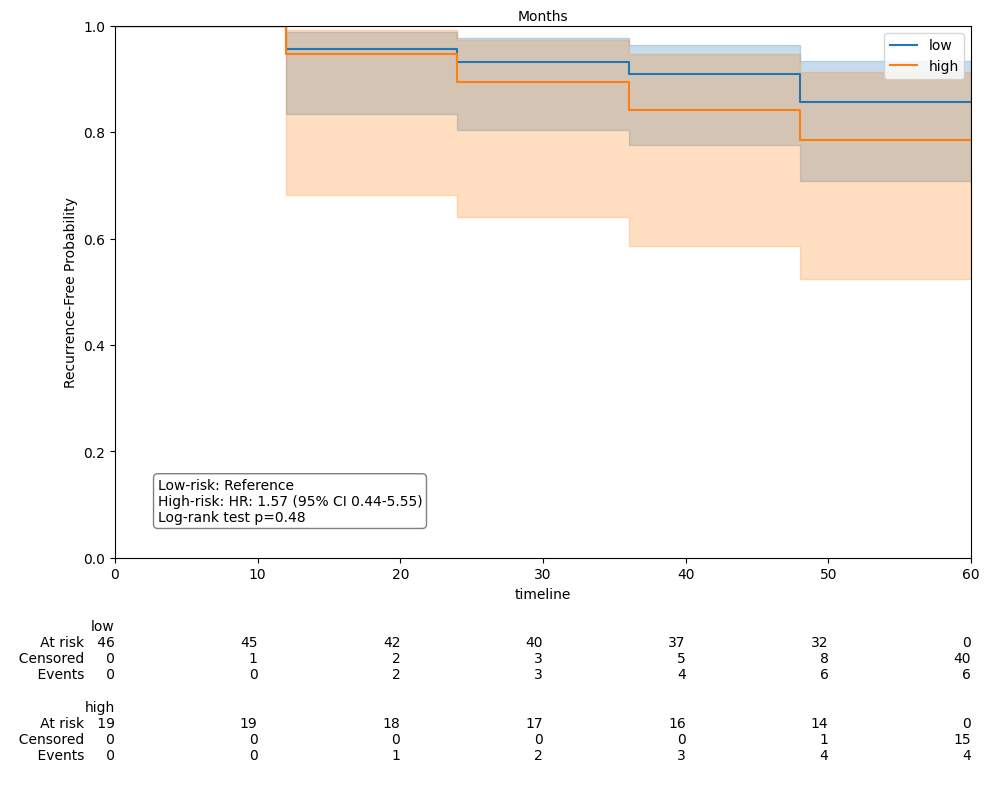** |
| **b**  Split 1 | **g**  Split 1 |
| 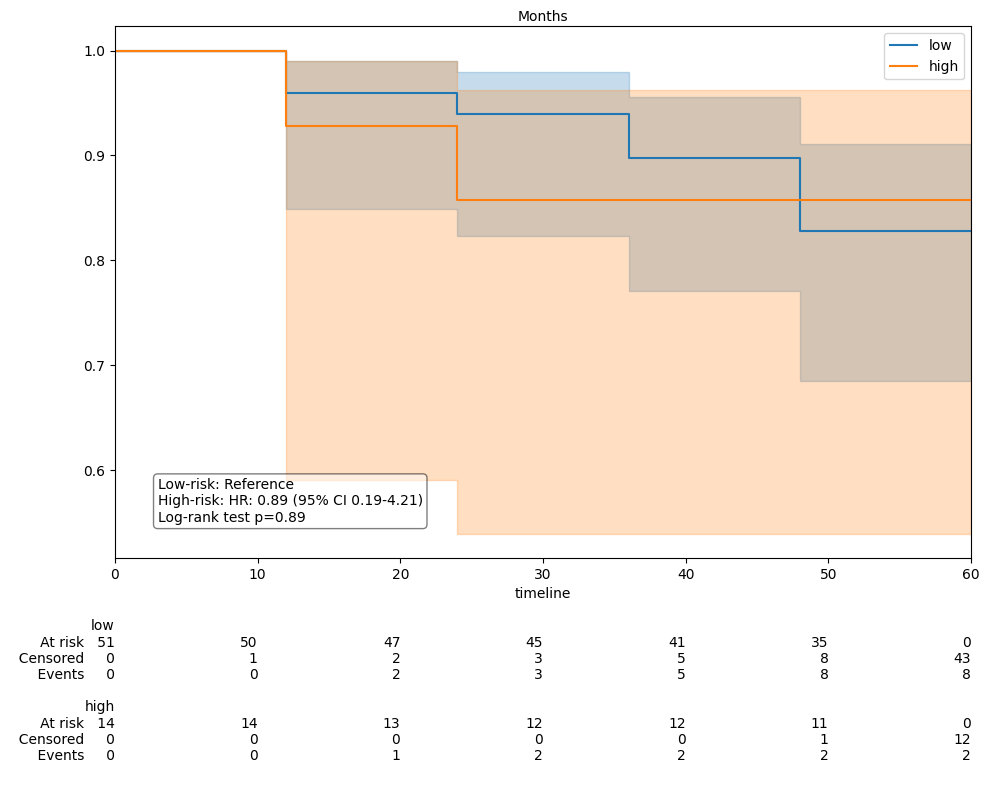 | 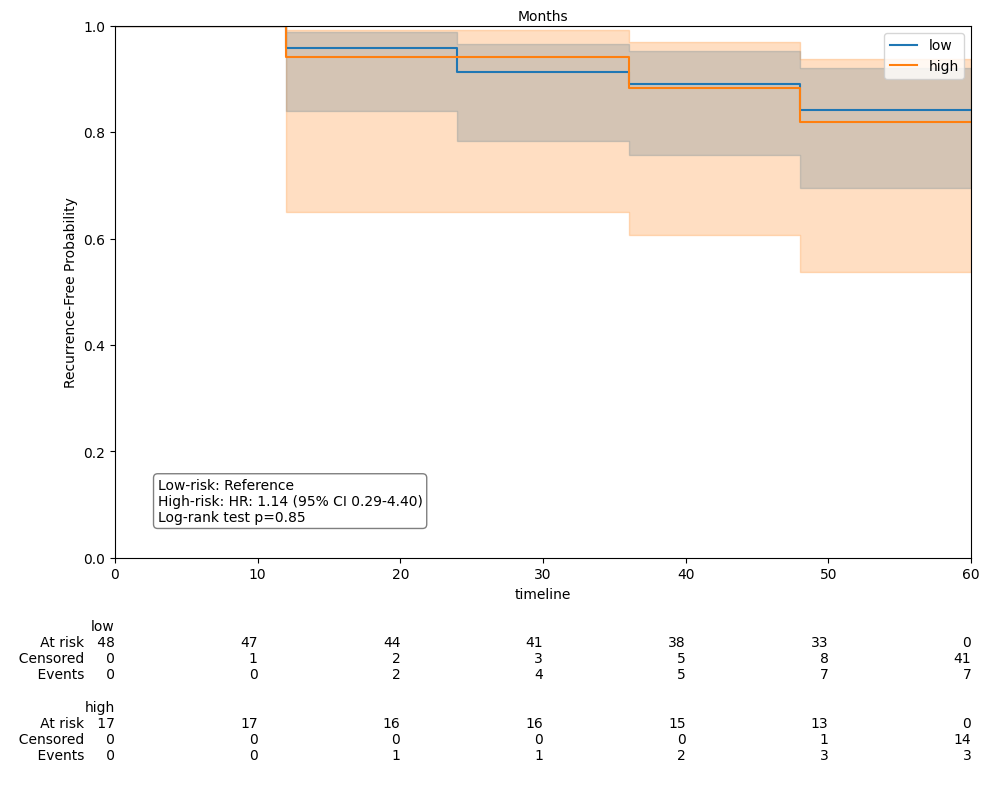 |
| **c** Split 2 | **h**  Split 2 |
| **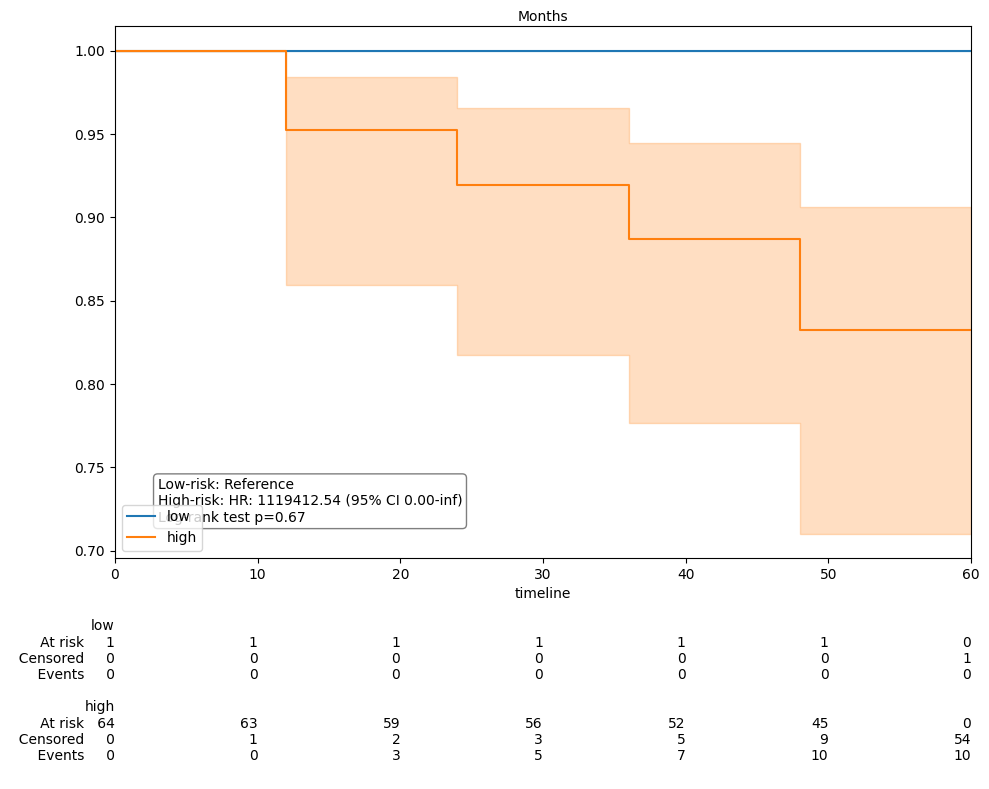** | 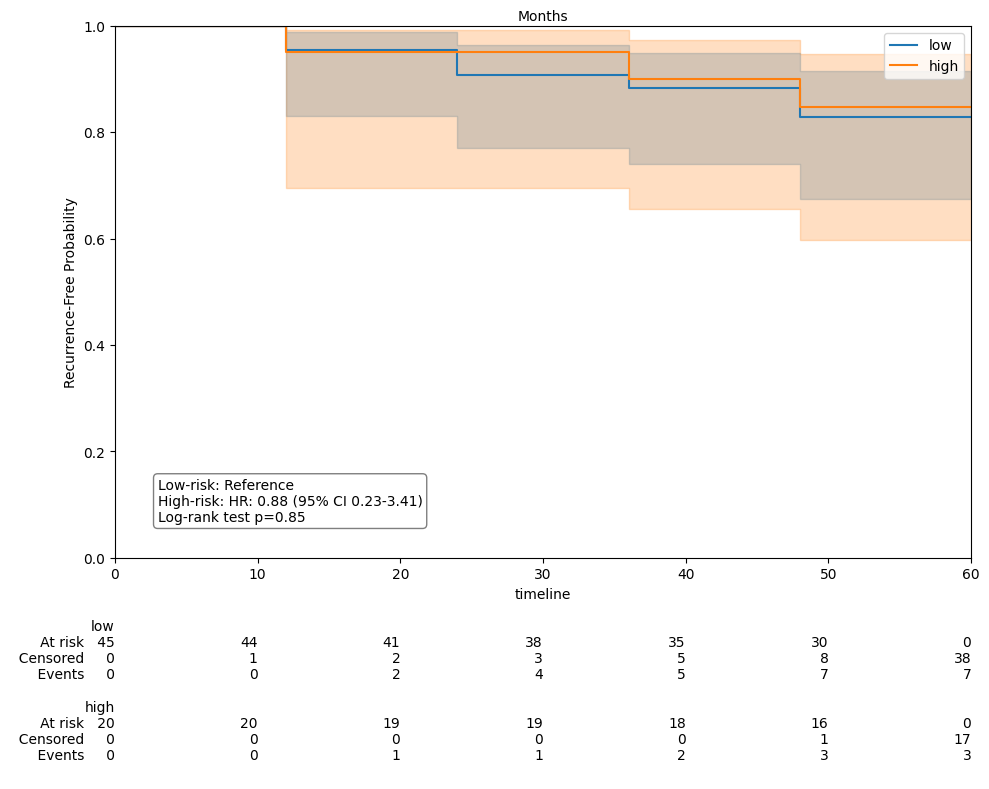 |
| **d**  Split 3 | **i**  Split 3 |
| NaN | 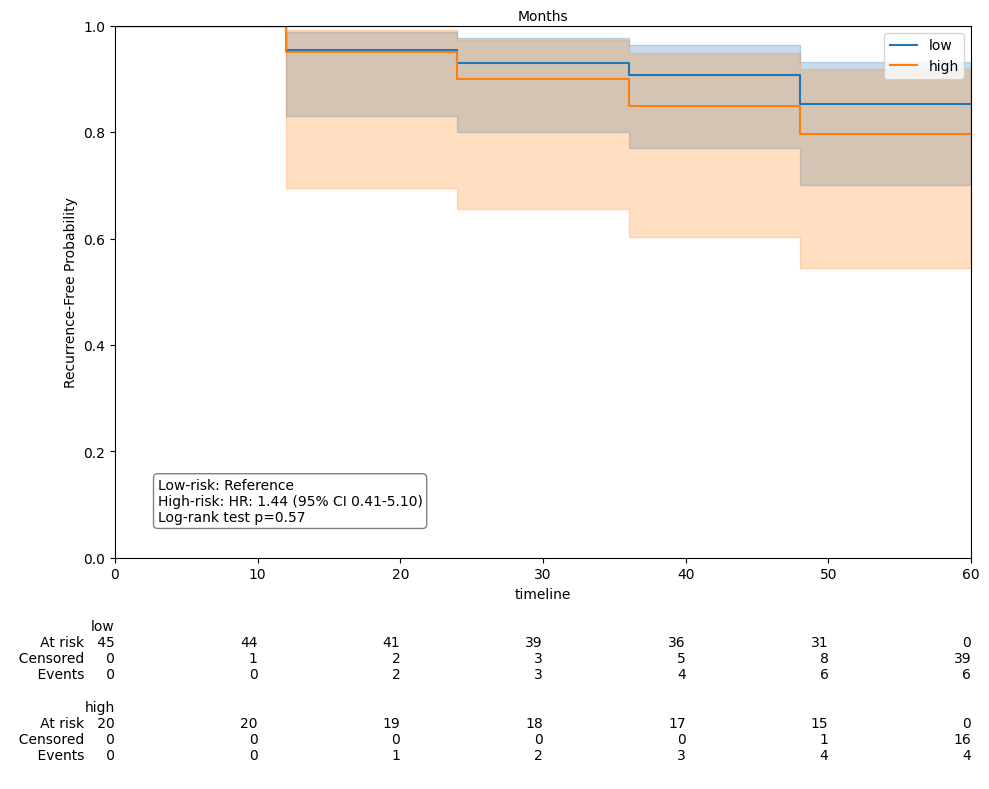 |
| **e**  Split 4 | **j**  Split 4 |
| **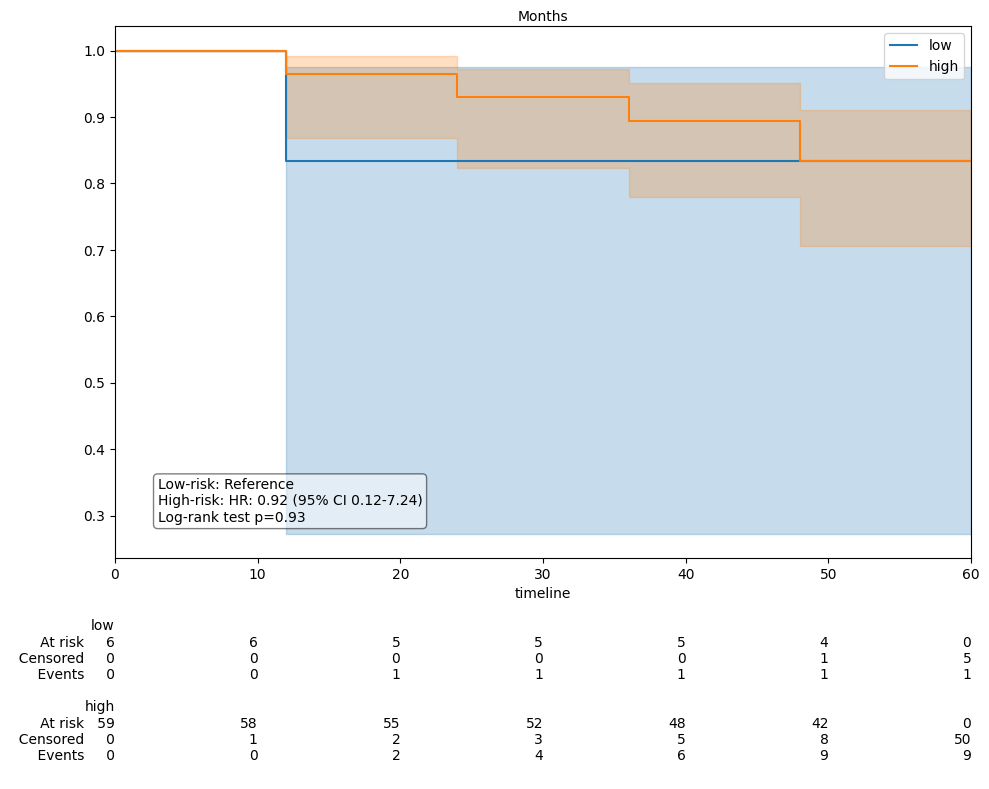** | 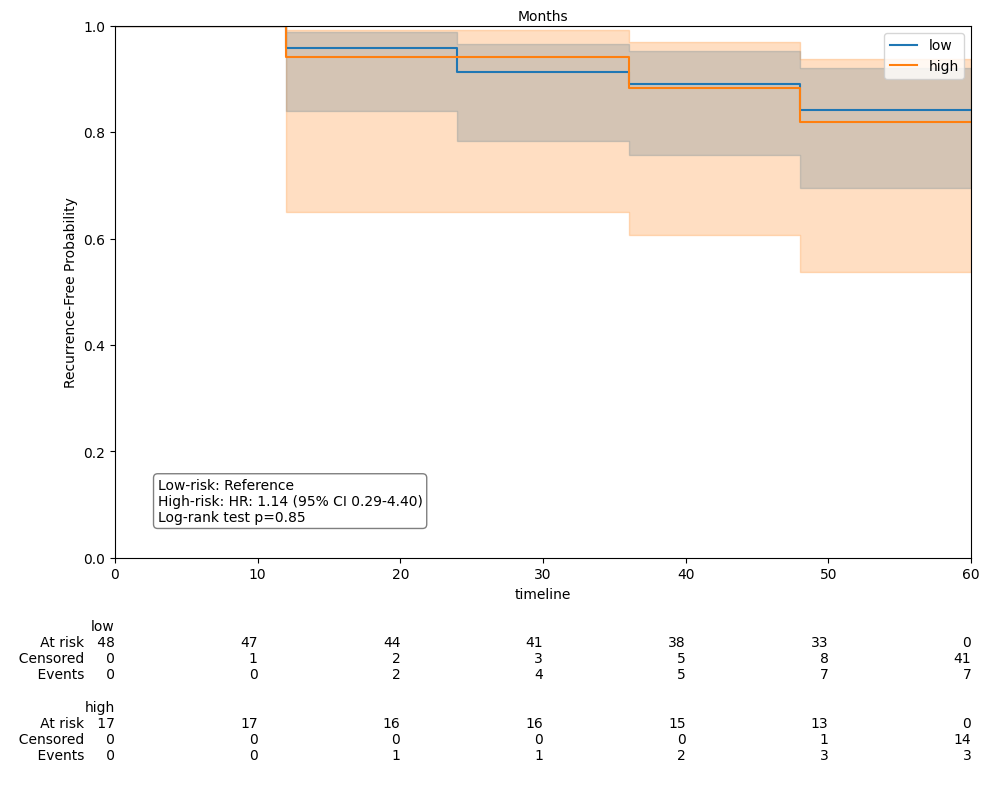 |

**Figure S3. Kaplan-Meier curves for models evaluated on 5-year follow-up in the Sloane test cohort.** (A-E). Integrative model; (F-J). Clinical-basic model. No KM curves are shown for the image-only models, because it only predicted one risk group. The shaded area refers to 95% confidence intervals. Abbreviations: low: predicted low-risk group; high: predicted high-risk group; HR: hazard ratio.
