# Supplementary material for: Deep learning for predicting invasive recurrence of ductal carcinoma in situ: leveraging histopathology images and clinical features
Source: eBioMedicine. 2025 May 28;116:105750. doi: 10.1016/j.ebiom.2025.105750 (PMC12162051; doi:10.1016/j.ebiom.2025.105750)
Supplement: Precision Consortium Members [file mmc2.docx]

| **First names** | **Surnames** |
| --- | --- |
| Jelle | Wesseling |
| Jos | Jonkers |
| Jacco | van Rheenen |
| Esther H. | Lips |
| Marjanka | Schmidt |
| Lodewyk F.A. | Wessels |
| Proteeti | Bhattacharjee |
| Alastair | Thompson |
| Serena | Nik-Zainal |
| Helen | Davies |
| Elinor J. | Sawyer |
| Andrew | Futreal |
| Nicholas | Navin |
| E. Shelley | Hwang |
| Fariba | Behbod |
| Daniel | Rea |
| Hilary | Stobart |
| Deborah | Collyar |
| Donna | Pinto |
| Ellen | Verschuur |
| Marja | van Oirsouw |
